# Targeted urine metabolomics for enhanced diagnosis of hypoxic-ischemic encephalopathy in newborns: A pilot study

**DOI:** 10.1371/journal.pone.0361619

**Published:** 2026-11-10

**Authors:** Darryl J. Adamko, Maryam Alyari, Sibasis Daspal, Kim Davis, Jae Newton, Salah Almubarek, Anas El-Aneed

**Affiliations:** 1 Department of Pediatrics, College of Medicine, University of Saskatchewan, Saskatoon, Saskatchewan, Canada; 2 College of Nutrition & Pharmacology, University of Saskatchewan, Saskatoon, Saskatchewan, Canada; University Medical Centre Ljubljana (UMCL) / Faculty of Medicine, University Ljubljana (FM, UL), SLOVENIA

## Abstract

While therapeutic hypothermia (cooling treatment) is successful in preventing hypoxic ischemic encephalopathy (HIE), current diagnosis is limited to clinical features and serum lactate. Many newborns at risk of HIE are not identified within the therapeutic window and consequently miss the opportunity for cooling treatment. We hypothesized that metabolomic analysis could more accurately identify infants at risk of HIE compared to other commonly used techniques, such as serum lactate. Newborns admitted for therapeutic hypothermia for HIE, were recruited (n = 11) and compared to four healthy infants. A targeted liquid chromatography-tandem mass spectrometry (LC-MS/MS) platform was used to measure metabolites in urine samples. We identified 15 metabolites that differentiated infants with HIE from healthy controls. The metabolomic signature was subsequently utilized to blindly and accurately re-identify the same infants 3 days later, after clinical recovery, with 100% accuracy, as their metabolic profiles resembled those of non-hypoxic healthy infants. The diagnostic performance of LC-MS/MS was superior to that of lactate alone. Urine metabolomic profiling has the potential to more accurately identify neonates at risk of long-term neurologic injury than lactate alone.

## Introduction

Birth of an infant is a physiologically stressful event, and a certain degree of hypoxemia is common. Unfortunately, some deliveries are more difficult with more hypoxemia causing end-organ damage. When the brain is affected, this is referred to as hypoxic-ischemic encephalopathy (HIE), a clinical syndrome characterized by symptoms such as abnormal tone and seizures. Current neuroprotective therapies aimed at preventing HIE are effective but must be applied within a relatively short window of time [1]. However, methods for diagnosing newborns at risk for HIE and long-term neurologic injury are limited. These methods include Apgar scores, serum acid-base deficits, serum lactate levels, electroencephalography (EEG) recordings, and magnetic resonance imaging (MRI) [2]. While each of these tests provides valuable information that can help predict severe outcomes of neonatal hypoxia, none in isolation offer the sensitivity and specificity necessary to guide preventative treatments for all at risk newborns [2–7]. As a result, many newborns experience significant hypoxia, but do not receive preventative therapy, leading to unfavorable long-term neurologic outcomes (i.e., cerebral palsy). There is a clinical need for a new methodology capable of providing an early diagnosis, to be used alongside those currently available (i.e., clinical assessment, metabolic and lactic acidosis, and aEEG) to assess a neonate’s hypoxic state, ultimately improving clinical outcomes [2,8].

Metabolomics using mass spectrometry (MS) provides an opportunity to investigate biochemical changes in response to disease states and/or injuries. Using a neonatal piglet model of HIE, we previously found that urine metabolomic analysis identified hypoxia with greater accuracy than lactate alone [9]. Since then, we have validated a targeted liquid chromatography-tandem mass spectrometry (LC-MS/MS) platform that accurately quantifies 39 metabolites in urine from a variety of metabolic pathways [10–13]. We hypothesized that this platform could be used in humans and would more accurately identify infants at risk of HIE compared to lactate alone. Using this platform, we established a potential metabolomic profile for neonatal HIE. Our ultimate objective is to develop this into a new clinical test to prevent HIE is all newborns.

## Materials and methods

This study was approved by our Institutional Review Board (IRB)/Biomedical Ethics Board at the University of Saskatchewan (Bio 17−116), and parents gave written consent. Infants were recruited from 2018-01-23–2024-07-11. Eleven newborns at risk of HIE were recruited from the NICU at Jim Pattison Children’s hospital, Saskatoon, SK, Canada, from January 2018 to December 2020 (Table 1). Criteria for enrollment included newborns who met the criteria for initiation of the whole body hypothermia therapy protocol based on the Canadian society of pediatrics clinical criteria [14]. Healthy infants were recruited from the normal newborn nursery within 6 hours of delivery from 2023-01-01–2024-07-03. Exclusion criteria included newborns <36 weeks gestation, those who did not pass urine within six hours of life, those with anomalies involving multiple organs, severe intra-uterine growth retardation (IUGR), and evidence of other systemic illness (e.g., infection, or metabolic disorders).

**Table 1:** Clinical characteristics.

Clinical Data	Subjects	
	Healthy	Risk of HIE
**Gender (Female / Male)**	2 Female / 2 Male	7 Female / 4 Male
**Average gestational Age (weeks)**	39.5 (38–41)	38 (37–40)
**Average birth Weight (kg)**	Normal	3.2 (2.33–4.31)
**Average Labor duration (Hrs)**	Did not record	5.5 []1 [–]10 []
**Type of delivery**	SVD*	SVD 3 / CS** 7
**Apgar scores**	Normal	2/1–4/5–6/10
**Time of 1**^**st**^ **urine sample hours from birth**	< 6 hours	4.6 (2–5.5)
**Average 2**^**nd**^ **urine sample collection**	None taken	47.8 (42–57) hours from birth
**Average 3**^**rd**^ **urine sample collection**	None taken	91 (88–95) hours from birth
**Average venous blood gas at birth**	None taken	pH: 7.04 (6.87–7.24)

*SVD: Spontaneous Vaginal delivery.

** CS: Caesarean Section.

### Urine sample collection and preparation

As per guidelines, therapeutic hypothermia requires 72 hours. We thought it was important to document the metabolomic data over this time. Urine samples were collected using a bag or urinary catheter. For newborns receiving therapy, three urine samples were collected; one at presentation to the NICU (within six hours of life), the next midway in the cooling period day 2 (47.8 hours SEM 1.29) and the last on day 3–4 (91 hours SEM 0.75) after receiving hypothermia therapy. Healthy infant urine was collected by a urine bag (n = 4). The urine was then promptly frozen at –20°C, and then stored for longer term at –80°C.

### Materials and chemicals

Most chemicals were purchased from Sigma-Aldrich (Oakville, ON) unless otherwise stated. Formic acid was from VWR (Edmonton, AB), ^12^C-2–4-(Dimethylamino) phenacyl (DmPA) bromide was from Combi-Blocks Inc (San Diego, CA), and β-Hydroxyisovaleric acid was from Alfa Aesar (Ward Hill, MA). Optima^TM^ LC-MS grade acetonitrile (ACN), water, methanol, and ammonium formate were purchased from Fisher Scientific (ON, CA). Sarcosine (99.4%) was purchased from Santa Cruz Biotechnology (Santa Cruz, CA, USA). Creatine (CRE) was purchased from Spectrum Laboratories Inc. (CA, USA). 1-methylnicotinamide-d3 chloride, choline-d9 chloride, creatine-d3 monohydrate, glucose-d2 (GLC-IS), guanidine acetic acid-d2 (GAAIS) and uracil-d2 (URC-IS) were purchased from C/D/N Isotope Inc. (QC, CA). Pyruvic acid-^13^C3 sodium salt was obtained from Omicron Biochemical Inc. (IN, USA).

### Instrumentation for targeted analysis

All samples were analyzed using an AB SCIEX 6500 QTRAP® instrument, a hybrid triple quadrupole-linear ion trap (QqQ-LIT) mass spectrometer equipped with an electrospray ionization source and linked to and Agilent 1290 Infinity UPLC system (ON, Canada). The detailed instrument setting for each method can be found in our prior publications [10–13].

### Dansylation labeling reaction

^12^C- and ^13^C-dansyl chloride, used as derivatizing reagents, were synthesized following the two-step protocol reported previously [10,11]. Light-chain ^12^C-dansyl chloride was used to label individual urine samples and the standard mix to prepare calibration curve samples. ^13^C-dansyl chloride was used to label the standard mix for the preparation of internal standards. Analyte standard stock solutions (3 mg/mL in 50% ACN) were aliquoted and pooled to generate a standard working stock solution mixture containing underivatized analytes with concentrations ranging from 12.5 to 160 mg/mL. Individual urine samples were subjected to a 2-fold dilution with ACN, vortexed, and centrifuged at 14,000 rpm for 10 minutes. The labeling of samples was performed according to the stablished method [10]. Briefly, stock solution or individual urine samples (50 μL) were mixed with 30 μL of sodium carbonate/sodium bicarbonate buffer (0.5M, pH 9.4). Next, 40 μL of ^12^C-dansyl chloride (10.13 mg/mL in ACN) was added, vortexing the mixture for 10–15 s. The reaction for one hour occurred in a 40∘C water bath. The reaction was quenched by adding 10 μL of 0.25M NaOH, followed by further heating at 60∘C for 10 min. To neutralize the medium, 70 μL of formic acid (300 nM in 50% ACN) was added. The same procedure was used to prepare internal standard by labelling the working stock solution using ^13^C-dansyl chloride. To prepare the calibration curve samples and QC samples, the ^12^C-dansylated working stock solution was diluted in 50% ACN to a volume of 40 µL. This solution was then spiked with 60 µL of blank surrogate urine matrix, which contained 10 mL internal standard. The derivatized urine samples were further diluted by adding 50 µL of 50% ACN containing 10 µL internal standard. Agilent 1290 Series ultra-high performance liquid chromatography (UHPLC) System with a Kinetex C18 column (2.6 μm, 100Å, 50 × 2.1 mm), part number 00B-4462-AN were used. Mobile phases were: A 0.1% formic acid in 5% acetonitrile (ACN); and B 0.1% formic acid in ACN. The gradient profile for metabolite separation was as follows: t = 0 min; 92% A, t = 1 min; 92% A, t = 3.25 min; 85% A, t = 6.15 min; 30% A, t = 6.25 min; 30% A, t = 6.35 min; 92% A, t = 7.35 min; 92% A. The flow rate was at 0.7 mL/min, and the column temperature was maintained at 22∘C.

### DmPA labeling reaction

^13^C-DmPA was synthesized following the protocol reported previously [11,12]. ^12^C-DmPA was purchased commercially from Combi-Blocks Inc. Light-chain ^12^C-DmPA was used to label individual urine samples and the standard mix to prepare standard curve samples while ^13^C-DmPA was used to label the standard mix for preparing internal standards. Aliquots of the analyte standard stock solutions (5 mg/mL for most analytes, and 10 mg/mL for lactic acid and Trigonelline) were pooled to generate a standard working stock solution mixture containing underivatized analytes. Individual urine samples were subjected to a 4-fold dilution with ACN, vortexed, and centrifuged at 14,000 rpm for 10 minutes. The DmPA derivatization method was previously published [12]. Briefly, 100 μL of a stock standard mixture or urine samples was mixed with a 100 μL of TEA solution (20 μL/mL in ACN). The mixture was vortexed followed by adding a 100 μL of ^12^C- or ^13^C-DmPA solution (30 mg/mL in ACN); the mixture was then vortexed, and spun down. The resulting mixture was heated in a water bath at 85∘C for 30 min. After derivatization, the solution was cooled to room temperature and diluted with 300 μL of formic acid solution (20 μL/mL in water). To prepare the calibration curve and QC samples, the ^12^C-DmPA labeled working stock solution was diluted in a 1:1 volumetric mixture of ACN:water (14:1) and formic acid solution (300 nM in water). Subsequently, 60 µL of this solution was spiked with 15 µL of underivatized pooled urine sample, which contained 9 µL internal standard. The derivatized urine samples were further diluted by adding 15 µL of 80% ACN containing 9 µL internal standard.

Liquid chromatography was performed with the same column as above, a Kinetex C18 column (2.6 μm, 100Å, 50 × 2.1 mm), part number 00B-4462-AN. Mobile phase A consisted of 5mM ammonium formate, 5% ACN, and 0.1% formic acid while mobile phase B consisted of 60% ACN, 40% methanol, and 0.1% formic acid. The gradient profile for metabolite separation was as follows: t = 0 min; 95% A, t = 0.25 min; 95% A, t = 1.25 min; 80% A, t = 2.25 min; 75% A, t = 4.25 min; 20% A, t = 5.15 min; 95% A, t = 9.15 min; 95% A. The flow rate was set at 0.7 mL/min and the column was maintained at 20 °C.

### HILIC-MS/MS method

The working stock and internal standard solutions were prepared separately from individual metabolite and internal standard stocks, respectively, as detailed in our past published work [13]. To prepare the calibration curve samples, aliquots of the working stock solution were serially diluted with water. For each calibration standard, 15 μL of the appropriate diluted solution was mixed with 85 μL ACN containing 15 μL internal standard. Urine samples were diluted 3-fold with water. Subsequently, 15 μL of each diluted sample was mixed with 85 μL of an ACN: internal standard working solution (80:5). The resulting mixtures were centrifuged at 14000 rpm for 10 minutes to precipitate proteins.

Liquid chromatography was performed as previously published [13], using a SeQuant^®^ ZIC^®^-HILIC column (3.5 µm, 100Å, 150 × 2.1 mm), part number 1.50442.0001. Mobile phase consisted of (A) 10 mM ammonium formate (AMFA) and (B) 0.1% formic acid (FA) in ACN: 10 mM AMFA (95: 5). The gradient profile for metabolite separation was as follows: t = 0 min; 20% A, t = 7 min; 30% A, t = 9 min; 50% A, t = 12 min; 50% A, t = 12.50 min; 20% A, t = 15 min; 20% A. At min 8, the flow was diverted, through an integrated switching valve, into waste in order to decrease the contamination of the ion source. Flow rate was set at 250 mL/min and the column was maintained at 55∘C.

All methods included the required quality control (QC) samples, in accordance with regulatory guidelines for clinical sample analysis, to ensure the accuracy of the quantitative data.

### Metabolomic data analysis

The LC-MS platforms allowed for the quantitative analysis of 39 metabolites using validated analytical methods, detailed in the methods section. To account for variations in the hydration status of study subjects, each metabolite concentration was normalized to the infant’s urinary creatinine. Creatinine concentrations in individual samples were determined using Jaffe’s colorimetric reaction with the QuantiChrom™ Creatinine Assay Kit (QC, CA).

Orthogonal partial least squares discriminant analysis (OPLS-DA) was performed using SIMCA-P 18.0 (Umetrics, USA). This analysis evaluates the relationship between the response vector Y (i.e., control versus HIE groups) and the matrix X (metabolite concentrations) by projecting both Y and X spaces onto a shared plane. Seven-fold internal cross-validation was applied. OPLS-DA generates a prediction score (ranging from 0 to 1) for each infant based on metabolite values as scores <0.5 predict healthy controls, while >0.5 indicate HIE state. This approach highlights the metabolites that differ significantly in terms of their concentrations between groups. However, most metabolites show minimal variation across groups. On the other hand, metabolites with consistent and substantial differences are considered more important for predicting patient state. Metabolites of low significance reduce model accuracy and were excluded. After analysis, only 15 metabolites were applied in the blinded analysis for HIE infants during and after therapeutic hypothermia treatment.

MetaboAnalyst 6.0 was used for pathway analysis, particularly the pathway topology module. The 15 significant metabolites identified from the OPLS-DA model were input into the software and matched with entries in the Human Metabolome Database (HMDB). Homo sapiens pathway library was selected, and the relative betweenness centrality metric was used for pathway topology analysis. MetaboAnalyst integrates multiple databases, including the Kyoto Encyclopedia of Genes and Genomes (KEGG) and HMDB, to identify possible pathways associated with a given condition [15].

## Results and discussion

### Subject characteristics

Eleven newborns with HIE were investigated. Seven females and four males. Average gestational age was 38 with birth weight between 2.33 to 4.31 kg (Table 1).

MS metabolomic analysis identifies significant differences in the urine of HIE infants compared to healthy infants.

Based on the concentrations, OPLS-DA generated a separation model between hypoxemic (n = 11) and healthy controls (n = 4). Many metabolites did not differ significantly between groups, as expected in such analysis. Including low-importance metabolites in the model reduces its accuracy; therefore, they were omitted in subsequent analysis. To improve the model and remove unnecessary metabolites, we analyzed the urine samples from the same HIE infants 2 days later, after therapeutic hypothermia treatment (n = 11), which were not included in the model. Low-significance metabolites were removed as much as possible while preserving the model’s ability to accurately classify non-hypoxic healthy infants and non-hypoxic infants recovered by day 3. The final OPLS-DA model accurately classified all blinded infants post-therapy as non-hypoxic with 100% accuracy (OPLS-DA prediction score < 0.5; 11 of 11 infants; Fig 1). Using these metabolites, we also analyzed the concentrations from HIE infants on day 2 of recovery, which were not used in the initial modeling (Fig 1). These metabolites suggested an intermediate metabolic phenotype between HIE and healthy infants.

**Fig 1:**
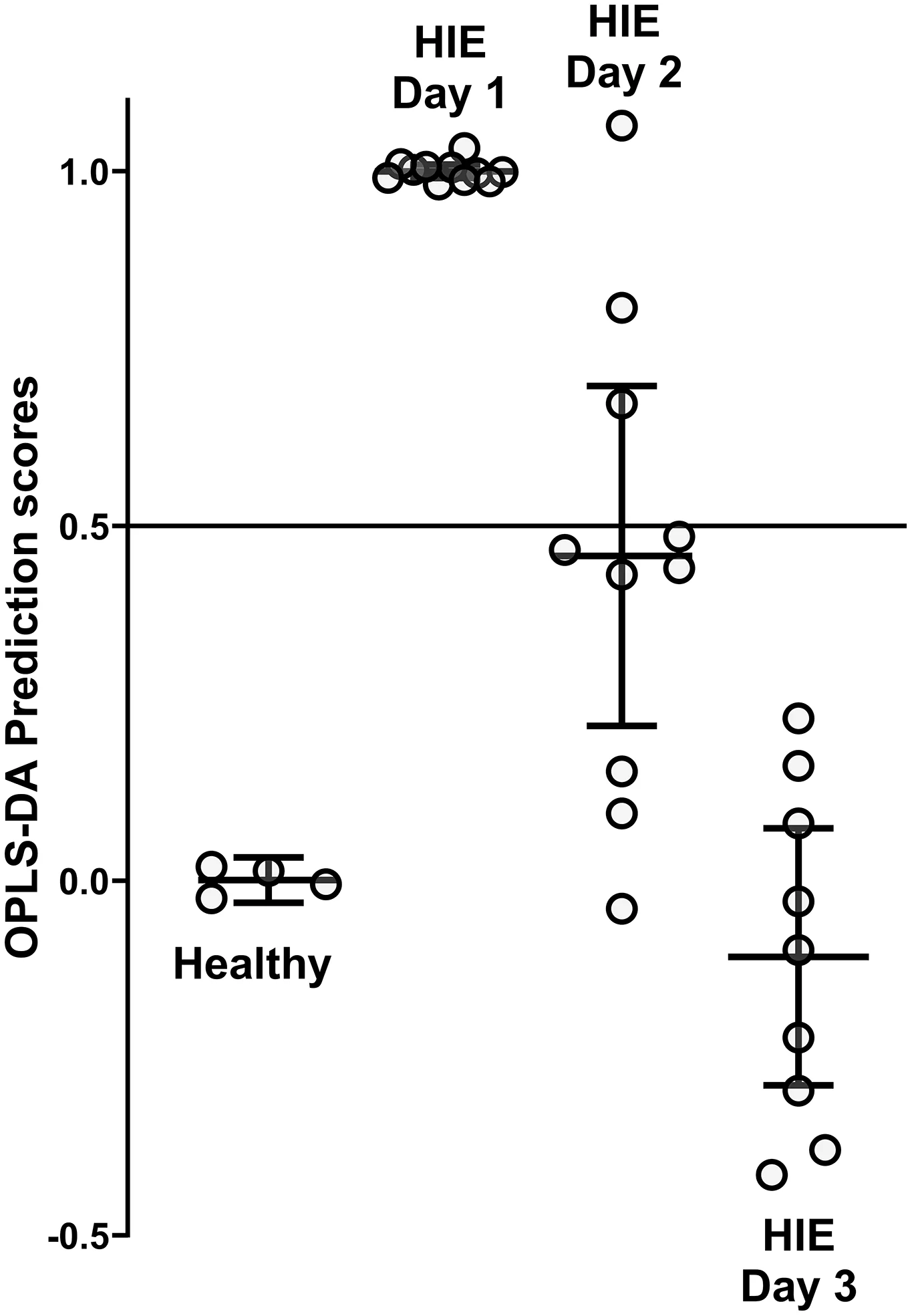
Differentiating hypoxic from non-hypoxic infants: The OPLS-DA algorithm separates data into distinct groups based on a score range of 0-1. A score closer to 0 indicates no hypoxemia (n = 4), while a score above 0.5 indicates hypoxemia (n = 11). The figure shows the prediction scores for each infant, with error bars representing the medians and interquartile ranges.

The final OPLS-DA model for separating HIE from non-hypoxic infants used 15 metabolites and seven components (R2 = 0.99 and Q2 = 0.97). The variations in metabolite concentrations among infant groups are shown in the Coefficient of Variation Plot (Fig 2A), while the Variable Importance Plot (Fig 2B) illustrated the importance of the metabolites in terms of their order.

**Fig 2:**
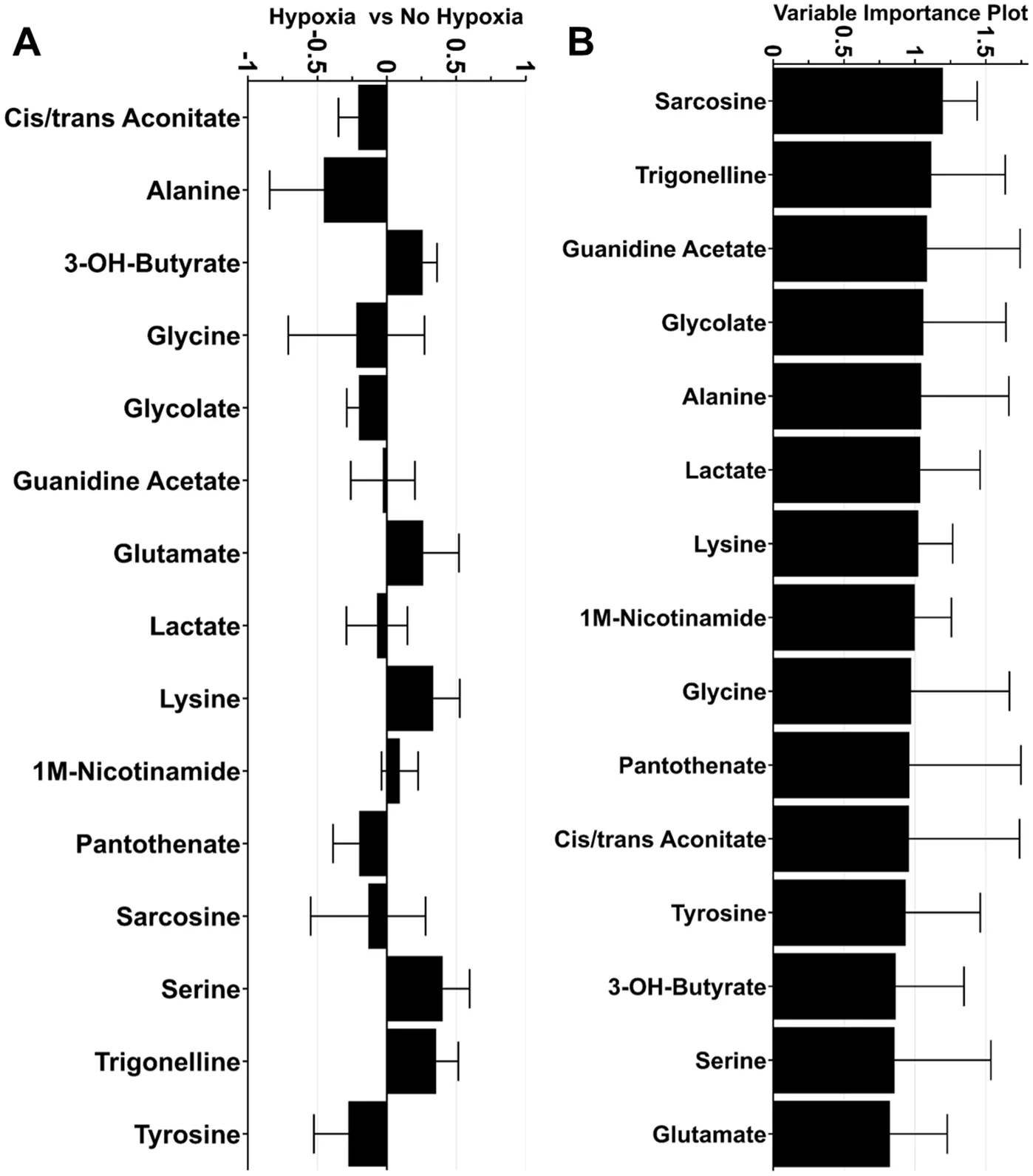
The final OPLS-DA model separating HIE from non-hypoxic infants. (A) Coefficients of Variation plot displaying scaled and centered metabolite differences between the groups; (B) Variable Importance Plot ranking the metabolites based on their significance in the model.

### Using lactate alone is less accurate compared to the metabolomic model

Finally, we determined the predictive performance of a model using lactate as a single variable (Fig 3). As expected, the model had less predictive value (R^2^ = 0.29 and Q^2^ = 0.21), with some HIE infants misclassified.

**Fig 3:**
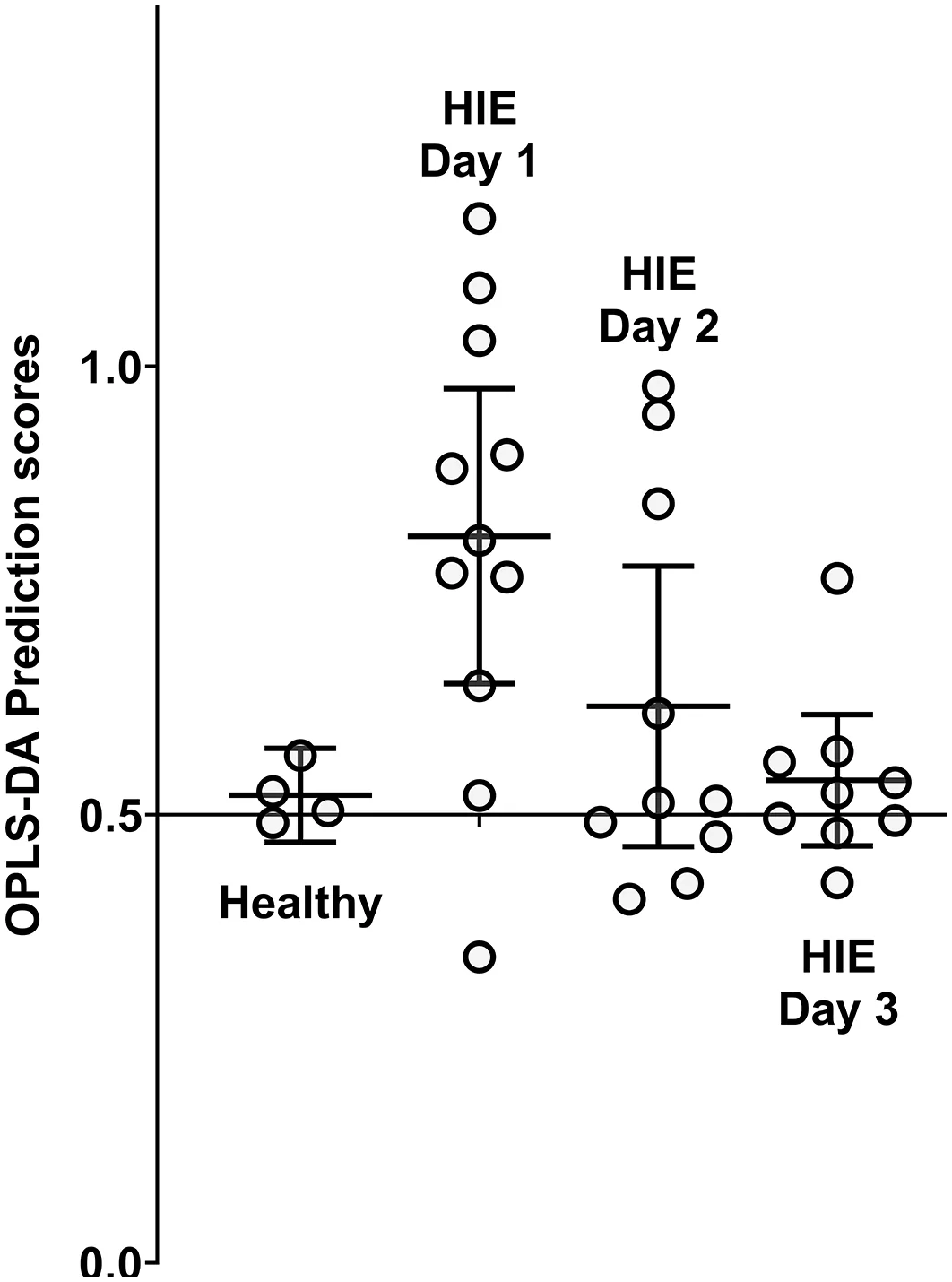
Using lactate alone is less accurate compared to the metabolomic model: The OPLS-DA algorithm assigns scores from 0 to 1, where values near 0 indicate no hypoxemia (n = 4) and values above 0.5 indicate hypoxemia (n = 11). Prediction scores for each infant are shown; error bars represent medians and interquartile ranges.

Determining whether to start a neuroprotective therapeutic intervention remains a challenge using current diagnostic tools. Apgar scores are variable and subject to the interpretation of the caregiver assessing for neonatal asphyxia [16]. This variability may be influenced by several factors, including maternal sedation or analgesia, as well as cardiovascular or neurological underlying conditions in the newborn [17]. Although lactate levels in the serum and base deficits can positively predict newborn patients who have experienced severe hypoxia, these measures are unreliable for detecting mild-to- moderate hypoxia and cannot rule out other medical conditions, such as septicemia [2,18,19]. Prediction of developmental delay typically relies on neurological examinations and additional tests, such as brain MRI [20,21] or EEG recordings [22,23]. However, a recent evaluation of EEG uses within 6 hours of hypoxic birth found that it failed to reliably predict long-term outcomes such as disability or death [24]. While MRI is an established tool for assessing hypoxic brain injury in neonates, it requires anesthesia and is unable to detect early hypoxic changes among infants [20,21]. The limitations in current standard diagnostic tests create a significant delay in the ability of physicians to diagnose patients in a timely manner while at the same time assessing the extent of hypoxia in newborns. An ideal diagnostic tool would be non-invasive, readily available by the NICU, which can provide a timely and accurate assessment of the newborn’s medical condition. LC-MS urine metabolomics of neonates at risk of HIE offers a novel and potentially more accurate diagnostic approach. Hospital-based LC-MS laboratories are already operational in many hospitals in North America, and sample analysis can be performed, in theory, as rapidly as current lactate testing. Importantly, this method is both faster and more accurate than EEG or MRI.

Unlike measuring one variable, namely lactate concentration, metabolomics allows for the assessment of multiple biomarkers that impact various metabolic pathways. In this study, a panel of 15 metabolites were identified that could be utilized to diagnose infants at risk of HIE. Consistent with our previous NMR analysis of urine in fetal piglet animal models of hypoxia, three key metabolites were identified, namely lactate, 1-methylnicotinamide, and alanine [9]. Additionally, our LC-MS analysis uniquely identified the following additional metabolites: cis/trans-aconitate, alanine, 3-hydroxybutyrate, glycine, glycolate, glutamate, guanidoacetic acid, lactate, lysine, 1-methylnicotinamide, pantothenate, sarcosine, serine, trigonelline, and tyrosine. MetaboAnalyst 6.0 was used to determine potential metabolic pathways. Key pathways were phenylalanine, tyrosine and tryptophan biosynthesis; glyoxylate and dicarboxylate metabolism; glycine, serine and threonine metabolism; and alanine, aspartate and glutamate metabolism; among others, as shown in Fig 4 and Table 2. Seven metabolites were not part of these pathways; however, they remain highly important for the predictive model and may contribute to the development of a diagnostic tool in the future. A previous study using MS analysis of urine saw similar findings including the change of the metabolome back to a healthy state over the recovery period [25]. There were differences in their reported metabolites from ours, as they used an untargeted MS approach without normalization for hydration.

**Table 2:** Summary of the most significant metabolic pathways identified using MetaboAnalyst.

Label Number	Metabolites	Common Metabolic Pathway Pathway	Total Metabolites in pathway	Impact	Holm adjusted p-value
1	Serine, Glycine, Guanidinoacetate	Glycine, serine and threonine metabolism	33	0.50	1.64E-01
2	Tyrosine	Phenylalanine, tyrosine and tryptophan biosynthesis	4	0.50	1.00E + 00
3	Alanine, Glutamate	Alanine, aspartate and glutamate metabolism	28	0.20	1.00E + 00
4	cis-Aconitate, Serine, Glycine, Glutamate	Glyoxylate and dicarboxylate metabolism	32	0.17	7.08E-03
5		Tyrosine metabolism	42	0.14	1.00E + 00
6	1-Methylnicotinamide	Nicotinate and nicotinamide metabolism	15	0.14	1.00E + 00
7	Tyrosine	Arginine biosynthesis	14	0.12	1.00E + 00
8	Glycine, Glutamate	Glutathione metabolism	28	0.11	1.00E + 00

**Fig 4:**
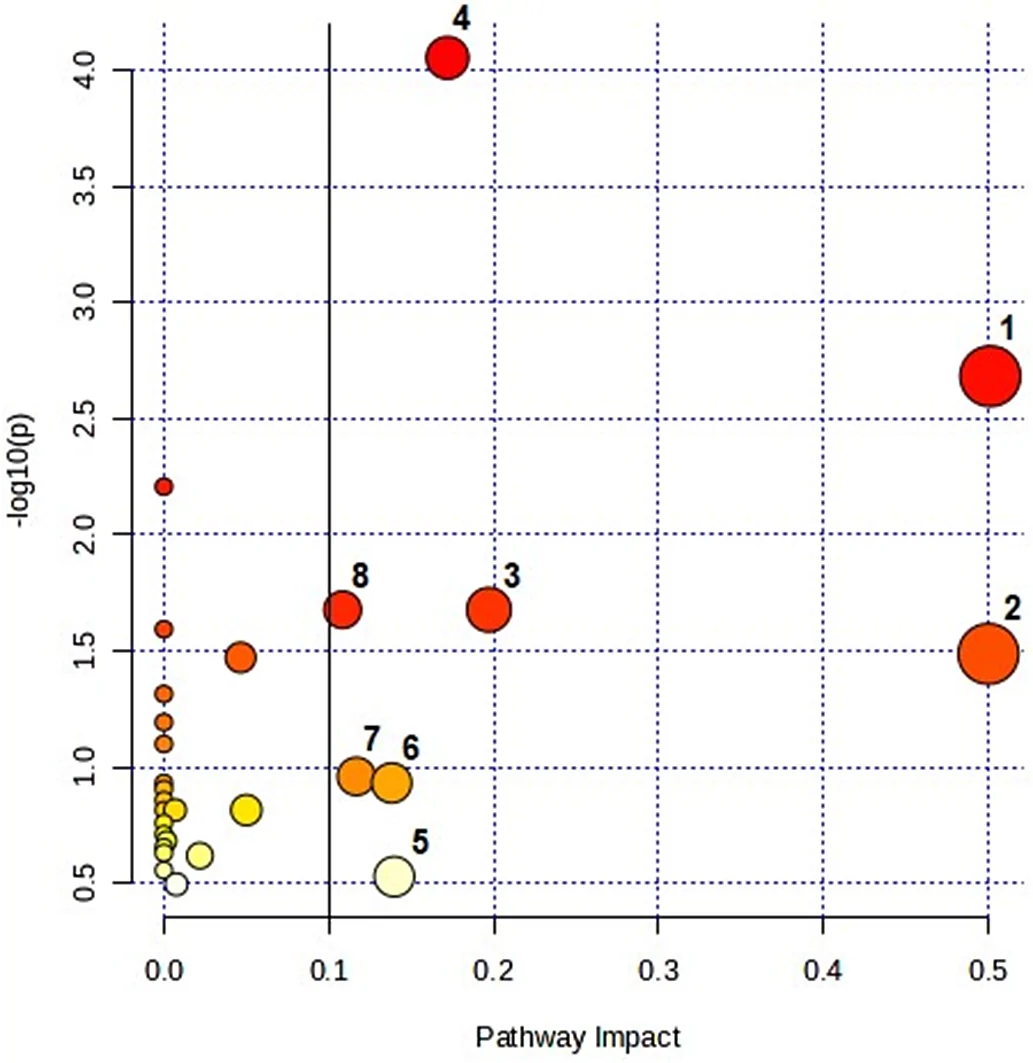
Pathway analysis of the significant metabolites performed using MetaboAnalyst 6.0. Circles represent metabolic pathways potentially involved in group separation. Numbers correspond to the pathways listed in Table 2.

The metabolites of importance in our study (Fig 2) are associated with cellular energy levels and metabolism, which is expected to be based on HIE pathophysiology. The findings in Fig 2 and Table 2 align to a large extent with existing literature, as outlined below. Lactate, a well-known product of anaerobic respiration, is an established marker of neonatal hypoxia in early stages [26]. Related to lactate, lysine degradation is reported in HIE pathophysiology [27] and in reports of HIE infants [28]. Levels of aromatic amino acids like tyrosine and alanine, often shift significantly in response to hypoxia, indicating metabolic disruptions [29].. Alanine levels, as reported, increase in the cerebral spinal fluid of fetal sheep during a hypoxic episode, remaining at elevated levels for up to two hours following the episode before gradually decreasing [30]. Similarly, glycine is elevated in the brains of HIE animal models [28,31].

During metabolic processes, glycine can convert to glyoxylate, which is subsequently transformed into glycolate, a metabolite that plays a critical role during hypoxia [32]. Hypoxia also significantly increases glutamate secretion, a key factor contributing to neuroexcitotoxicity [33]. Additionally, alterations in ketone bodies like 3-hydroxybutyrate have been reported in the plasma of HIE infants [34], which may result from induced hypometabolic hypothermia or altered substrate mobilization and utilization. While not previously reported for brain injury, some of the metabolites identified (Fig 2) have been associated with kidney injury. Aconitate levels are known to change in response to hypoxic injury in the kidneys [35], and trigonelline is also well described with kidney failure [36]. Similarly, serine, tyrosine, pantothenate and lysine are recognized markers of kidney injury [37,38].

The above discussed metabolites highlight the impact that hypoxia can exert on cellular metabolism throughout the body, which could be relevant when considering therapeutic hypothermia. Kidney injury may be easier to detect and could serve as an early indicator of the risk of brain injury. The remaining identified metabolites were most likely a result of either inflammation or amino acid dysregulation; both of which are directly related to hypoxia. 1-Methylnicotinamide (1MNT) possess anti-inflammatory properties and is a metabolite of nicotinamide [39], and there is promising evidence suggesting that treatments with 1MNT offer neuroprotection in neonatal animal models of hypoxia-ischemia [40]. Sarcosine may reduce oxidative stress, potentially helping to protect the brain from damage in rats [41].

It should be noted that no one metabolite can diagnose all infants accurately. For example, while lactate is critical in the model, it was not significantly elevated in some hypoxic infants. Our data suggests that accurate diagnosis requires the measurement of multiple variables (i.e., metabolites) and targeted metabolomics provides this capability.

We acknowledge there are limitations for this study. Firstly, the infants do vary in weight, and this could have unforeseen effects on the metabolomic profile. Certainly, the caloric intake of a healthy infant vs an HIE infant in the NICU at days 1–3 will be different, but there is no way to avoid this. In addition, the sample size for this study is small, especially for the healthy infants. It is not easy to get a urine sample on a healthy newborn; most mothers just want to go home with their newborn. The HIE number is also low, but relatively balanced to its comparator. The metabolomic profile in this study will be validated in larger cohorts of neonates. The end goal is to develop a point of care diagnostic tool that is based on the reported metabolomic profile in the urine. The use of urine, a body fluid that is non-invasively collected, makes the test suitable for non-tertiary care settings where most deliveries often occur. The analysis of a first urine sample can provide insights and guide clinical decision making for obstetricians and family doctors performing deliveries.
