# Supplementary material for: Targeted urine metabolomics for enhanced diagnosis of hypoxic-ischemic encephalopathy in newborns: A pilot study
Source: PLoS One. 2026 Nov 10;21(11):e0361619. doi: 10.1371/journal.pone.0361619 (PMC13750575; doi:10.1371/journal.pone.0361619)
Supplement: Supplementary file 1 [file pone.0361619.s002.docx]

**PONE-D-25-25931**

**Targeted Urine Metabolomics for Enhanced Diagnosis of Hypoxic-Ischemic Encephalopathy in Newborns**

**PLOS ONE

Dear Dr. Adamko,

Thank you for submitting your manuscript to PLOS ONE. After careful consideration, we feel that it has merit but does not fully meet PLOS ONE’s publication criteria as it currently stands. Therefore, we invite you to submit a revised version of the manuscript that addresses the points raised during the review process.**

- **From my point of view I agree very much with the reviewer, hovwever I miss date and number of approved study from the Ethics committe. The authors should explain why from collected samples of urine elapsed many years before they collected urine samples for control group of patiients.**

*Response: We added the Ethics number at line 98. Regarding the time difference, we have addressed this in a response below. We struggled with getting EEG data on healthy babies, and eventually decided to abandon the idea to at least report the urine data we did have.*

- **References are missing on urine metabolomics in infants with HIE already published and which are of relevance.**

*Response: We have addressed each Reviewer below.*

**Demographic data of maters and children are scarce, no informations whether induced hypothermia means wholew body hypothermia or only hypothermia of the head, ...**

*Response: The point of the study was to capture urine metabolomic data on infants at high risk of HIE. These were on infants enrolled for whole body cooling therapy. We did not capture data on the mothers as this was small pilot study, and it wouldn’t necessarily influence the metabolomic data. The exclusion data are also there to lessen the need for data from the mother. We added that to the Methods line 73.*

**Kind regards**

**Please submit your revised manuscript by Oct 30 2025 11:59PM. If you will need more time than this to complete your revisions, please reply to this message or contact the journal office at** [**plosone@plos.org**](mailto:plosone@plos.org)**. When you're ready to submit your revision, log on to https://www.editorialmanager.com/pone/ and select the 'Submissions Needing Revision' folder to locate your manuscript file.

Please include the following items when submitting your revised manuscript:**

- **A rebuttal letter that responds to each point raised by the academic editor and reviewer(s). You should upload this letter as a separate file labeled 'Response to Reviewers'.**
- **A marked-up copy of your manuscript that highlights changes made to the original version. You should upload this as a separate file labeled 'Revised Manuscript with Track Changes'.**
- **An unmarked version of your revised paper without tracked changes. You should upload this as a separate file labeled 'Manuscript'.**

**If you would like to make changes to your financial disclosure, please include your updated statement in your cover letter. Guidelines for resubmitting your figure files are available below the reviewer comments at the end of this letter.

If applicable, we recommend that you deposit your laboratory protocols in protocols.io to enhance the reproducibility of your results. Protocols.io assigns your protocol its own identifier (DOI) so that it can be cited independently in the future. For instructions see:** [**https://journals.plos.org/plosone/s/submission-guidelines#loc-laboratory-protocols**](https://can01.safelinks.protection.outlook.com/?url=https%3A%2F%2Ftrack.editorialmanager.com%2FCL0%2Fhttps%3A%252F%252Fjournals.plos.org%252Fplosone%252Fs%252Fsubmission-guidelines%2523loc-laboratory-protocols%2F1%2F010f01994cf920b2-ca3e8221-e86f-4242-af11-2c3508f59c2f-000000%2F8h7oy3lAaySRwjxds49ajHBVNwTVIWbeYJGCFXfeuVQ%3D228&data=05%7C02%7Cdarryl.adamko%40usask.ca%7Cf44fcd34288c415b718b08ddf444bba7%7C24ab6cd0487e47229bc3da9c4232776c%7C0%7C0%7C638935300457465326%7CUnknown%7CTWFpbGZsb3d8eyJFbXB0eU1hcGkiOnRydWUsIlYiOiIwLjAuMDAwMCIsIlAiOiJXaW4zMiIsIkFOIjoiTWFpbCIsIldUIjoyfQ%3D%3D%7C0%7C%7C%7C&sdata=ZRHZlRrgkrmD6bC01lCU6lAGt81VT2BqemEWyU8jpW0%3D&reserved=0)**. Additionally, PLOS ONE offers an option for publishing peer-reviewed Lab Protocol articles, which describe protocols hosted on protocols.io. Read more information on sharing protocols at** [**https://plos.org/protocols?utm_medium=editorial-email&utm_source=authorletters&utm_campaign=protocols**](https://can01.safelinks.protection.outlook.com/?url=https%3A%2F%2Ftrack.editorialmanager.com%2FCL0%2Fhttps%3A%252F%252Fplos.org%252Fprotocols%253Futm_medium%3Deditorial-email%2526utm_source%3Dauthorletters%2526utm_campaign%3Dprotocols%2F1%2F010f01994cf920b2-ca3e8221-e86f-4242-af11-2c3508f59c2f-000000%2FsYi36cLUB-28VO13OBGYUJ0XwQq1bDgYhOaaNjFmESc%3D228&data=05%7C02%7Cdarryl.adamko%40usask.ca%7Cf44fcd34288c415b718b08ddf444bba7%7C24ab6cd0487e47229bc3da9c4232776c%7C0%7C0%7C638935300457489355%7CUnknown%7CTWFpbGZsb3d8eyJFbXB0eU1hcGkiOnRydWUsIlYiOiIwLjAuMDAwMCIsIlAiOiJXaW4zMiIsIkFOIjoiTWFpbCIsIldUIjoyfQ%3D%3D%7C0%7C%7C%7C&sdata=U%2FQHhIX1F7Wwow4KrbaxRrtEeRHY8fDUqLEWsvMFV6g%3D&reserved=0)**.

We look forward to receiving your revised manuscript.**

**Kind regards,**

**Stefan Grosek, Ph.D., M.D.,
Academic Editor
PLOS ONE**

**Journal Requirements:**

**When submitting your revision, we need you to address these additional requirements.**

**1. Please ensure that your manuscript meets PLOS ONE's style requirements, including those for file naming. The PLOS ONE style templates can be found at**

<https://journals.plos.org/plosone/s/file?id=wjVg/PLOSOne_formatting_sample_main_body.pdf> and 
<https://journals.plos.org/plosone/s/file?id=ba62/PLOSOne_formatting_sample_title_authors_affiliations.pdf>

*Response: Corrected. Thank you*

**2. We note that the grant information you provided in the ‘Funding Information’ and ‘Financial Disclosure’ sections do not match.**

**When you resubmit, please ensure that you provide the correct grant numbers for the awards you received for your study in the ‘Funding Information’ section.**

*Response: We are adding grant numbers from Children's Hospital Foundation of Saskatchewan 416661 and 419327, a CIHR Catalyst grant G00020476, and SHRF 416111. Please remove the NSERC grant.*

**3. Thank you for stating the following in your manuscript:**

**“The authors thank the Jim Pattison Children’s Hospital Foundation. Additional support has been through NSERC, CIHR and SHRF.”**

**We note that you have provided funding information that is not currently declared in your Funding Statement. However, funding information should not appear in the Acknowledgments section or other areas of your manuscript. We will only publish funding information present in the Funding Statement section of the online submission form.**

**Please remove any funding-related text from the manuscript and let us know how you would like to update your Funding Statement. Currently, your Funding Statement reads as follows:**

**“The author(s) received no specific funding for this work.”**

**Please include your amended statements within your cover letter; we will change the online submission form on your behalf.**

*Response: This study was funded; sorry for the confusion. We have removed the funding text in the manuscript and have added the granting agencies above. Thank you for correcting the online area.*

**4. We note that your Data Availability Statement is currently as follows:**

**“All relevant data are within the manuscript and its Supporting Information files.”**

**Please confirm at this time whether or not your submission contains all raw data required to replicate the results of your study. Authors must share the “minimal data set” for their submission. PLOS defines the minimal data set to consist of the data required to replicate all study findings reported in the article, as well as related metadata and methods (https://journals.plos.org/plosone/s/data-availability#loc-minimal-data-set-definition).**

**For example, authors should submit the following data:**

**- The values behind the means, standard deviations and other measures reported;
- The values used to build graphs;
- The points extracted from images for analysis.**

**Authors do not need to submit their entire data set if only a portion of the data was used in the reported study.**

**If your submission does not contain these data, please either upload them as Supporting Information files or deposit them to a stable, public repository and provide us with the relevant URLs, DOIs, or accession numbers. For a list of recommended repositories, please see** [**https://journals.plos.org/plosone/s/recommended-repositories**](https://journals.plos.org/plosone/s/recommended-repositories)**.**

**If there are ethical or legal restrictions on sharing a de-identified data set, please explain them in detail (e.g., data contain potentially sensitive information, data are owned by a third-party organization, etc.) and who has imposed them (e.g., an ethics committee). Please also provide contact information for a data access committee, ethics committee, or other institutional body to which data requests may be sent. If data are owned by a third party, please indicate how others may request data access.**

*Response: We see that the Reviewers both responded in the affirmative when asked the question #3, “Have the authors made all data underlying the findings in their manuscript fully available?”. As such, we are not sure what level of data the Editor is requesting. In addition, we are obligated by our Health Region and the Local Ethics Board to keep all raw data in the trust of the University of Saskatchewan. I’m not sure what level of data you require. For example, each infant had a measured level of a metabolite. If I put that into a public repository for every infant another researcher could steal that and use it for any purpose; could they not? I am happy to share data with another researcher if they wanted to contact us as a collaborator. As such we prefer to put that as a statement.*

**5. When completing the data availability statement of the submission form, you indicated that you will make your data available on acceptance. We strongly recommend all authors decide on a data sharing plan before acceptance, as the process can be lengthy and hold up publication timelines. Please note that, though access restrictions are acceptable now, your entire data will need to be made freely accessible if your manuscript is accepted for publication. This policy applies to all data except where public deposition would breach compliance with the protocol approved by your research ethics board. If you are unable to adhere to our open data policy, please kindly revise your statement to explain your reasoning and we will seek the editor's input on an exemption. Please be assured that, once you have provided your new statement, the assessment of your exemption will not hold up the peer review process.**

*Response: As above, we do not put all the data for our infants into a public repository. We are happy to collaborate with researchers and share data based on formal arrangements.*

**6. Your ethics statement should only appear in the Methods section of your manuscript. If your ethics statement is written in any section besides the Methods, please delete it from any other section.**

*Response: Removed. Thank you*

**7. Please include a copy of Table 3 which you refer to in your text on pages 14 and 19.**

*Response: There are only 2 tables, and there is no table three. This was an error on our part, and I have changed table 2 to 3 in the text.*

**8. If the reviewer comments include a recommendation to cite specific previously published works, please review and evaluate these publications to determine whether they are relevant and should be cited. There is no requirement to cite these works unless the editor has indicated otherwise.**

*Response: Agreed. Thank you*

**Additional Editor Comments:**
**Dear Authors
This is interesting sstudy reviewed by two reviewers who addressed many issues to be answered by the Authors, small number of control patients, lack ofsamples in control group, and so on. From my point of view I agree very much with the reviewer, hovwever I miss date and number of approved study from the Ethics committe. The authors should explain why from collected samples of urine elapsed many years befor they collected urine samples for control group of patiients.
Refrences are missing on urine metabolomics in infants with HIE already published and which are of relevance.
Demographic data of maters and children are scarce, no informations whether induced hypothermia means wholew body hypothermia or only hypothermia of the head, ...**
*Response: Please see our responses to each of the Reviewers below. Thank you.*

**Reviewers' comments:
Reviewer's Responses to Questions**

**Comments to the Author
1. Is the manuscript technically sound, and do the data support the conclusions?
The manuscript must describe a technically sound piece of scientific research with data that supports the conclusions. Experiments must have been conducted rigorously, with appropriate controls, replication, and sample sizes. The conclusions must be drawn appropriately based on the data presented.**

**Reviewer #1: Partly**

**Reviewer #2: Partly**

*Response: Thank you*

**2. Has the statistical analysis been performed appropriately and rigorously?**

**Reviewer #1: Yes**

**Reviewer #2: No**

*Response: We have responded to Reviewer 2 below.*

**3. Have the authors made all data underlying the findings in their manuscript fully available?

The** [**PLOS Data policy**](https://can01.safelinks.protection.outlook.com/?url=http%3A%2F%2Ftrack.editorialmanager.com%2FCL0%2Fhttp%3A%252F%252Fwww.plosone.org%252Fstatic%252Fpolicies.action%2523sharing%2F1%2F010f01994cf920b2-ca3e8221-e86f-4242-af11-2c3508f59c2f-000000%2Fr5RZmLk1WfsbNOYZ5MCVi3jvciwKUyVCp1KhoraseD4%3D228&data=05%7C02%7Cdarryl.adamko%40usask.ca%7Cf44fcd34288c415b718b08ddf444bba7%7C24ab6cd0487e47229bc3da9c4232776c%7C0%7C0%7C638935300457504661%7CUnknown%7CTWFpbGZsb3d8eyJFbXB0eU1hcGkiOnRydWUsIlYiOiIwLjAuMDAwMCIsIlAiOiJXaW4zMiIsIkFOIjoiTWFpbCIsIldUIjoyfQ%3D%3D%7C0%7C%7C%7C&sdata=iEk1IDIAAgO5RdTKIuDfluCiXSMHUm9Hr1edLSOGVpY%3D&reserved=0) **requires authors to make all data underlying the findings described in their manuscript fully available without restriction, with rare exception (please refer to the Data Availability Statement in the manuscript PDF file). The data should be provided as part of the manuscript or its supporting information, or deposited to a public repository. For example, in addition to summary statistics, the data points behind means, medians and variance measures should be available. If there are restrictions on publicly sharing data—e.g. participant privacy or use of data from a third party—those must be specified.**

**Reviewer #1: Yes**

**Reviewer #2: Yes**

*Response: Thank you*

**4. Is the manuscript presented in an intelligible fashion and written in standard English? PLOS ONE does not copyedit accepted manuscripts, so the language in submitted articles must be clear, correct, and unambiguous. Any typographical or grammatical errors should be corrected at revision, so please note any specific errors here.**

**Reviewer #1: No**

**Reviewer #2: Yes**

*Response: We have responded to Reviewer #1 below. Thank you*

**5. Review Comments to the Author

Please use the space provided to explain your answers to the questions above. You may also include additional comments for the author, including concerns about dual publication, research ethics, or publication ethics. (Please upload your review as an attachment if it exceeds 20,000 characters)**

**Reviewer #1: The study of et Darryl al. is very interesting and potentially usefull for neonatal practice. Nevertheless there are important issues that have to be resolved before publication:**
*Response: Thank you*

**Comment 1: First of all the sample of the control patients is really to small: I don’t think OPLS-DA analysis are accurate for less that 5 determinations.**

*Response:* *We agree that the sample size for the control babies is small, and it is not ideal. Initially we were trying to get EEG data on both HIE and healthy infants. This was hard to coordinate in the NICU and became impossible in the healthy group. Most mothers just want to go home with their newborn. After many months of trying, we elected to just get urine alone without EEG in the healthy. It is not easy to get a urine sample on a healthy newborn. We first found mothers to consent, then hoped the infant could urinate in the bag within the expected time. Further, we could not ask a mother for a catheter urine sample, nor would Ethics allow such an invasive procedure. We lost some consented infants for this issue. The other Reviewer had a similar concern about sample size, and we agree this is a limitation. The HIE number is also low, but relatively balanced to its comparator. We hope that we can publish these important data with a revised title with the words “Pilot Study”. We have added text to the Discussion (lines 343-7) outlining the data as preliminary warranting a larger study. This work started as a small grant from our local Hospital foundation. We want to do this as a multicentre study, and this paper is expected to help justify the idea as a new grant application. OPLS-DA analysis can be done on this number of infants, especially when it is compared to a similarly smaller sample size of affected infants (n=11). In addition, the data was log transformed to meet test requirements. The number of metabolites we chose to identify HIE infants was 15, but that was chosen to achieve an ideal diagnostic result. We suspect with a higher number of infants the number of metabolites would decrease. Again, a larger study is needed. We hope the Reviewers can remain enthusiastic about these data and get us to the next phase of this important work.*

**Comment 2: Furthermore, In the study the eleven infants studied are classified as moderate or severe HIE. This score should be used to correctly assess the presence of HIE even if symptoms of HIE are absent or mild. The severe cases are easily identifiable. Should these patient be removed from the analysis as they may influence a score that is needed to identify potential patients that need to be treated despite having mild symptoms?**

*Response:* *There was a mistake in the table headers. Thank you for catching this error on our part. As stated in the Methods, “*Eleven newborns at risk of HIE were recruited from the NICU at Jim Pattison Children’s hospital, Saskatoon, SK, Canada, from January 2018 to December 2020 (Table 1.). Criteria for enrollment included newborns who met the criteria for initiation of the hypothermia therapy protocol based on the Canadian society of pediatrics clinical criteria. *We did not attempt to classify them as mild, moderate or severe for the metabolomic study. This table was created as part of the earlier study and was not continued. The mild case (n=1) was not analyzed for metabolomics. The objective was to confirm a metabolome that predicts the need for cooling therapy. As such, there are no severe patients to remove. In future, if we are successful in getting funds to do a larger clinical trial this idea to stratify severity of HIE could be considered. We have removed this infant from the table and simply re-labelled the Infants as Healthy or Risk of HIE, line 80, Table 1.*

**Comment 3: What is the goal of the study? This has to be added at the end of the introduction**

*Response: In the paper the last lines of the introduction state “We hypothesized that this platform could be used in humans, and would more accurately identify infants at risk of HIE compared to lactate alone. Using this platform, we established a metabolomic profile for neonatal HIE. We have added line 64-5, “Our ultimate objective is to develop this into a new clinical test to prevent HIE is all newborns.” We hope this satisfies the Reviewer. Thank you*

**Comment 4: The methodology that was followed is not clear, this chapter has to be written so that the reader can understand which metabolites have been tested
MS metabolomic analysis identifies significant differences in the urine of HIE infants compared to healthy infants. This paragraph is very confusing and has to be rewritten. For example are only the 15 significative metabolites being tested or all the 39?
“To refine the model, we analyzed the urine samples from the same HIE infants 2 days later, after therapeutic hypothermia treatment (n=11), which were not included in the model.” Sentence is not clear. Rephrase! “We also analyzed the concentrations from HIE infants on day 2 of recovery, which were not used in the initial modeling (Figure 1). These metabolites suggested an intermediate metabolic phenotype between HIE and healthy infants.” Hat does “HIE infants on day 2 of recovery” mean? The sentence is really difficult to understand**

*Response: We are sorry for the confusion. The metabolite number for the model was reduced to 15 from the original 39 total. In the Methods lines 203-8 we state, “*However, most metabolites show minimal variation across groups. On the other hand, metabolites with consistent and substantial differences are considered more important for predicting patient state. Metabolites of low significance reduce model accuracy and were excluded. After analysis, only 15 metabolites were applied in the blinded analysis for HIE infants during and after therapeutic hypothermia treatment”. This phrase was restated in lines 226-33. *We have changed the text at line 226, “To improve the model and remove unnecessary metabolites, we analyzed the urine samples from the same HIE infants 2 days later, after therapeutic hypothermia treatment (n=11),…..”. At line 232 we added the text, “Using these metabolites, “. We agree this can be confusing, and we hope the new text satisfies the Reviewer.*

**Comment 4: What is the value of testing again the patients that have undergone hypothermia? What do the authors want to prove? This is not clear in the text**

*Response:* *We thought it was important to document the time course and recovery from cooling therapy which lasts 72 hours. It is possible that the brain injury would create metabolites that persist and that could be relevant. Based on our data, the metabolites that signal HIE are becoming normalized similar to healthy at day 3. We believe this is relevant and have added text at line 85. Reviewer 2 has shown us a similar paper with improvement in the metabolome at day 3, and we have added that paper to this report. We hope the Reviewer agrees. Thank you*

**Comment 5: The last sentence of the conclusion has to be removed “The metabolomic-test data may also indicate the need for the involvement of a neonatologist”**

*Response: Removed. Thank you*

**Reviewer #2: The paper presents an interesting study based on urinary metabolomic analysis in a cohort of neonates with hypoxic-ischemic encephalopathy (HIE) and a small cohort of healthy controls, with the aim of identifying potential metabolites or metabolic pathways that may be pathognomonic of HIE.

Below are my observations for the Authors:
Comment 1: Lines 75–77: The authors correctly state that none of the methods cited, when considered individually, have predictive power with respect to HIE outcome. However, I would specify that the combination of aEEG, clinical evaluation, and metabolic acidosis may be more predictive than any of these parameters taken in isolation.**

*Response: We added the text “in isolation” to line 48. We hope this satisfies the Reviewer. Thank you*

**Comment 2: Lines 78–79: Rather than “more precise methods,” as the authors write, what is needed are methodologies** **capable of providing an early diagnosis, to be used alongside those currently available (clinical assessment, metabolic and lactic acidosis, and aEEG).**

*Response: We have added new text at line 52, “There is a clinical need for a new methodology capable of providing an early diagnosis, to be used alongside those currently available (i.e. clinical assessment, metabolic and lactic acidosis, and aEEG) more precise methods to assess a neonate’s hypoxic state, ultimately improving clinical outcomes”. Thank you*

**Comment 3: Line 248: The large variability in weight influences the variability of the metabolic snapshot. I would suggest describing the range of weight variability within the study limitations section.**

*Response: We have added new text in a limitations section. Line 340-2, “We acknowledge there are limitations for this study. Firstly, the infants do vary in weight, and this could have unforeseen effects on the metabolomic profile.” We hope this satisfies the Reviewer. Thank you*

**Comment 4: Lines 251–252: The authors do not provide a 1:1 match between HIE patients (n=11) and healthy controls (n=4). Furthermore, the average weight of the healthy controls is not specified. I am concerned that this limits the robustness of the findings, and I recommend addressing this issue in the limitations.** **The sample size is not large, and this should be acknowledged among the study’s limitations.**
*Response: As above, we have an expanded limitations section, lines 340-46. Thank you*

**Other remarks
Comment 5: Table 2: Why were the second and third urine samples not collected in healthy neonates? It would have been interesting and useful to compare the metabolic trajectory of healthy neonates with that of those affected by HIE.**

*Response: As discussed earlier, it would be very difficult in our opinion to get mothers of healthy infants to get urine samples on their infants for three days. These were healthy babies in a normal nursery. Most would be home and not in hospital by day 3.*

**Comment 6: Previous studies (including this, which I recommend to cite: Valerio E, Mardegan V, Stocchero M, Cavicchiolo ME, Pirillo P, Poloniato G, D'Onofrio G, Bonadies L, Giordano G, Baraldi E. Urinary metabotypes of newborns with perinatal asphyxia undergoing therapeutic hypothermia. PLoS One. 2022 Aug 16;17(8):e0273175) have shown that the metabolome of HIE neonates tends to converge with that of healthy neonates toward the end (and after the conclusion) of therapeutic hypothermia. However, without these corresponding timepoints in healthy neonates, this convergence is difficult to demonstrate in the present study.**
*Response: We were unaware of this report and have added it to the paper. It is important to note that they did not study a healthy infant cohort, but one with a normal MRI head. It is possible these infants did have a degree of HIE. They did not follow the infants to see if they were truly healthy long term (no cerebral palsy). In contrast we studied what would be considered the typical healthy infant. They also saw different metabolites. This could be due to the fact that they did not do targeted quantification of metabolites, but rather untargeted. Our method is more accurate. I addition, they did not account for hydration status with Creatinine. We did, and again our values are likely more accurate. We added some text to this effect at lines 294-97. Thank you*

**Comment 6: In the Methods section, it is unclear how the authors classified a subgroup of neonates with mild HIE. Is this subclassification truly useful? The key message to convey to readers should be that metabolomics can distinguish an HIE neonate (who therefore requires therapeutic hypothermia) from a non-HIE neonate (who does not). I am not convinced that the study provides sufficient evidence to suggest that metabolomics can also be used to grade HIE severity, particularly since this would not alter the clinical management (i.e., initiation of hypothermia).**

*Response: There was a mistake in the table headers. As stated in the Methods, “*Eleven newborns at risk of HIE were recruited from the NICU at Jim Pattison Children’s hospital, Saskatoon, SK, Canada, from January 2018 to December 2020 (Table 1.). Criteria for enrollment included newborns who met the criteria for initiation of the hypothermia therapy protocol based on the Canadian society of pediatrics clinical criteria. *We did not attempt to classify them as mild, moderate or severe for the metabolomic study. This table was part of an earlier study that was not continued. The mild case (n=1) was not analyzed for metabolomics. The objective was to confirm a metabolome that predicts the need for cooling therapy. We have removed this infant from the table and simply labelled the Table as Healthy and Risk of HIE, line 81. Thank you for catching this mistake and we agree with the Reviewer. Thank you.*

**Comment 7: The authors do not clarify how they classified the annotation levels of the metabolites. What level of confidence do they have that the 15 identified metabolites are indeed correctly annotated? Are they all level 1 annotations? Please specify.**

*Response: The idea that stating a metabolite is level 1 versus 3 is not really applicable to our work, nor does it really make a metabolite clinically relevant. This is an idea that is used in untargeted MS approaches. Our method does not need the level of annotation. It is targeted MS, which means we used standard curves for each metabolite, and the values are accurate within 5-10% similar to standard for a clinical lab. The OPLS-DA software (SIMCA) analyzing the data does not give us a level 1-3 value. It assumes the values are accurate. The metabolites chosen are based on blinded analysis and reassessment for correct clinical phenotyping. It is possible that some of the lesser important metabolites in that figure may prove to not be essential to a clinical test in future; that can only be known with larger studies with repeated clinical cohorts. We added the word “targeted” to the Abstract and in Methods line 105. We state this idea in the Discussion line 297. We hope the Reviewer understands our point and agrees. Thank you.*

**Comment 8: The data on lysine (see Fig. 2) are noteworthy. Intact lysine has been widely demonstrated to exert neuroprotective effects, both in animal models and in humans (as a reference: Valerio E, Stocchero M, Pirillo P, D'Errico I, Bonadies L, Galderisi A, Giordano G, Baraldi E. Neurosteroid pathway derangement in asphyctic infants treated with hypothermia: an untargeted metabolomic approach. EBioMedicine. 2023 Jun;92:104636. ). I would emphasize this aspect more strongly in the Discussion. Moreover, do the authors have evidence of increased lysine catabolites (e.g., pipecolic acid) in HIE neonates compared to non-HIE neonates?**

*Response: Thank you for this reference. That hypothesis may be important. We have added text at line 308-9. We did not measure pipecolic acid as it was not in our targeted platform. This is something for us to consider in future. Thank you.*

**Comment 9: The authors do not specify whether confounding factors that may interfere with metabolomic analysis (e.g., enteral/parenteral nutrition, plasma transfusions, antibiotic therapy) were present during sample collection, nor how these factors were adjusted for in the analysis. One of the major challenges of metabolomic analysis in neonates is precisely to disentangle the effects of the pathological process under investigation from those of such confounders. A comment from the authors on this point would be valuable.**

*Response: This is another limitation to note, and we have added this to the paragraph, line 342, “Certainly, the caloric intake of a healthy infant vs an HIE infant in the NICU at days 1-3 will be different, but there is no way to avoid this.” We hope this satisfies the Reviewer. Thank you.*
