# Supplementary material for: Targeted urine metabolomics for enhanced diagnosis of hypoxic-ischemic encephalopathy in newborns: A pilot study
Source: PLoS One. 2026 Nov 10;21(11):e0361619. doi: 10.1371/journal.pone.0361619 (PMC13750575; doi:10.1371/journal.pone.0361619)
Supplement: Supplementary file 2 [file pone.0361619.s003.docx]

Stefan Grosek, Ph.D., M.D.,
Academic Editor
PLOS One

PONE-D-25-25931R1

Targeted Urine Metabolomics for Enhanced Diagnosis of Hypoxic-Ischemic Encephalopathy in Newborns: a Pilot Study

PLOS One

Dear Dr. Grosek,

Thank you for the opportunity to submit a revised version of the manuscript using track changes and the point by point responses to the Reviewers below.

**Editor Comments:

Dear Authors

Despite much improvement in revised version of your article the third reviewer found some important issues to be addressed before final decision: The principal issue is that the reported “100% accuracy” does not appear to represent an independent validation: the day-3 samples from the same HIE infants were used during metabolite model refinement were also used as longitudinal samples subsequently described as a blinded test set. Given the very small dataset, the use of 15 metabolites and seven OPLS-DA components, there is a substantial risk of overfitting. The analysis would be more convincing with nested cross-validation/permutation testing and diagnostic measures such as sensitivity, specificity, and AUC with confidence intervals, followed ultimately by validation in an independent cohort.
A second concern is potential confounding: HIE samples were collected in 2018-2020 whereas healthy controls were recruited in 2023-2024, creating possible storage-duration, batch, and temporal differences that are not adequately addressed. Additionally, statements that the metabolomic approach is superior to lactate, more accurate than EEG/MRI, or can identify infants at risk of long-term neurologic injury are stronger than can be supported by this small pilot study without an independent validation cohort or long-term neurologic outcomes. I would therefore encourage further revision, particularly to tone down the conclusions and more explicitly separate the proof-of-concept results from validated diagnostic performance.**

Response: We understand the Editor’s points and have addressed them below to the specific Reviewer.

**Comments to the Author**

**1. If the authors have adequately addressed your comments raised in a previous round of review and you feel that this manuscript is now acceptable for publication, you may indicate that here to bypass the “Comments to the Author” section, enter your conflict of interest statement in the “Confidential to Editor” section, and submit your "Accept" recommendation.**

**Reviewer #2: All comments have been addressed**

Response: Thank you

**Reviewer #3: (No Response)**

**2. Is the manuscript technically sound, and do the data support the conclusions?

The manuscript must describe a technically sound piece of scientific research with data that supports the conclusions. Experiments must have been conducted rigorously, with appropriate controls, replication, and sample sizes. The conclusions must be drawn appropriately based on the data presented.**

**Reviewer #2: Yes**

**Reviewer #3: Partly**

Response: Thank you

**3. Has the statistical analysis been performed appropriately and rigorously?**

**Reviewer #2: Yes**

**Reviewer #3: Yes**

Response: Thank you

**4. Have the authors made all data underlying the findings in their manuscript fully available?

The** [**PLOS Data policy**](https://can01.safelinks.protection.outlook.com/?url=http%3A%2F%2Ftrack.editorialmanager.com%2FCL0%2Fhttp%3A%252F%252Fwww.plosone.org%252Fstatic%252Fpolicies.action%2523sharing%2F1%2F010f01a0c81df8a5-c5c292b2-6c5f-446d-8899-7ebadf63382b-000000%2FB7urKnh8uSR6fqSKsuCOILGI8sAbcl-lyP3MZrrrKDQ%3D258&data=05%7C02%7Cdarryl.adamko%40usask.ca%7C10951f69daa74f2860f908df19ac5ef4%7C24ab6cd0487e47229bc3da9c4232776c%7C0%7C0%7C639257900241932921%7CUnknown%7CTWFpbGZsb3d8eyJFbXB0eU1hcGkiOnRydWUsIlYiOiIwLjAuMDAwMCIsIlAiOiJXaW4zMiIsIkFOIjoiTWFpbCIsIldUIjoyfQ%3D%3D%7C0%7C%7C%7C&sdata=GFg2fMLGjnDOVaoj3AIggJ1yilaF751T%2B6uWROAL68w%3D&reserved=0) **requires authors to make all data underlying the findings described in their manuscript fully available without restriction, with rare exception (please refer to the Data Availability Statement in the manuscript PDF file). The data should be provided as part of the manuscript or its supporting information, or deposited to a public repository. For example, in addition to summary statistics, the data points behind means, medians and variance measures should be available. If there are restrictions on publicly sharing data—e.g. participant privacy or use of data from a third party—those must be specified.**

**Reviewer #2: Yes**

**Reviewer #3: Yes**

Response: Thank you

**5. Is the manuscript presented in an intelligible fashion and written in standard English?

PLOS ONE does not copyedit accepted manuscripts, so the language in submitted articles must be clear, correct, and unambiguous. Any typographical or grammatical errors should be corrected at revision, so please note any specific errors here.**

**Reviewer #2: Yes**

**Reviewer #3: Yes**

Response: Thank you

**6. Review Comments to the Author

Please use the space provided to explain your answers to the questions above. You may also include additional comments for the author, including concerns about dual publication, research ethics, or publication ethics. (Please upload your review as an attachment if it exceeds 20,000 characters)**

**Reviewer #2: (No Response)**

Response: Thank you

**Reviewer #3:**

1. **The revised manuscript is clearer and the authors have addressed several reviewer concerns, including clarification of the study objective, expansion of the limitations and addition of relevant prior literature.**

Response: Thank you

2. **However, important methodological concerns remain. The principal issue is that the reported “100% accuracy” does not appear to represent an independent validation: the day-3 samples from the same HIE infants were used during metabolite model refinement were also used as longitudinal samples subsequently described as a blinded test set. Given the very small dataset, the use of 15 metabolites and seven OPLS-DA components, there is a substantial risk of overfitting. The analysis would be more convincing with nested cross-validation/permutation testing and diagnostic measures such as sensitivity, specificity, and AUC with confidence intervals, followed ultimately by validation in an independent cohort.**

Response: We acknowledge this is a small data set. The study struggled to recruit because of the COVID outbreak in 2020. We have added that text to the Limitations section. We also agree that the “accuracy” statements need to be changed. The Day 3 samples we not truly blinded but instead represent a method to remove metabolites of low importance but also over-fitting (line 275). We agree that the goal is to avoid over-fitting. We have also added the Reviewer’s statistical suggestions for a future study in the Limitations section (line 393). We hope the Reviewer can now see the value of these data to publish. We are in the process of writing grants based on the pilot data and a publication is essential. Thank you.

3. **A second concern is potential confounding: HIE samples were collected in 2018-2020 whereas healthy controls were recruited in 2023-2024, creating possible storage-duration, batch, and temporal differences that are not adequately addressed.**

Response: The time delay occurred as a result of the COVID outbreak. We have stated this openly in the manuscript, and we have stated this as a limitation of the study in the Discussion line 388. We hope this will satisfy the Reviewer.

4. **Additionally, statements that the metabolomic approach is superior to lactate, more accurate than EEG/MRI, or can identify infants at risk of long-term neurologic injury are stronger than can be supported by this small pilot study without an independent validation cohort or long-term neurologic outcomes. I would therefore encourage further revision, particularly to tone down the conclusions and more explicitly separate the proof-of-concept results from validated diagnostic performance.**

Response: We agree with the Reviewer and we have made extensive changes in the text throughout the marked manuscript (Abstract, Introduction, and Discussion) to reflect the pilot nature of these data. We are not saying that this “is” superior to Lactate alone (or MRI/EEG), but this “could be”. We also have clearly stated the limitations in the Discussion as suggested. This paper should be interesting to clinicians. I hope we have satisfied the Reviewer with this text. Thank you.
