# Supplementary material for: Targeted urine metabolomics for enhanced diagnosis of hypoxic-ischemic encephalopathy in newborns: A pilot study
Source: PLoS One. 2026 Nov 10;21(11):e0361619. doi: 10.1371/journal.pone.0361619 (PMC13750575; doi:10.1371/journal.pone.0361619)
Supplement: Peer review history

# Peer review: Decision Letter 0

**DOI:** 10.1371/journal.pone.0361619.r001

**Authors:** Stefan Grosek

15 Sep 2025

Dear Dr. Adamko,

- From my point of view I agree very much with the reviewer, hovwever I miss date and number of approved study from the Ethics committe. The authors should explain why from collected samples of urine elapsed many years before they collected urine samples for control group of patiients.
- References are missing on urine metabolomics in infants with HIE already published and which are of relevance.
  Demographic data of maters and children are scarce, no informations whether induced hypothermia means wholew body hypothermia or only hypothermia of the head, ...
  Kind regards

Please submit your revised manuscript by Oct 30 2025 11:59PM. If you will need more time than this to complete your revisions, please reply to this message or contact the journal office at plosone@plos.org. When you're ready to submit your revision, log on to https://www.editorialmanager.com/pone/ and select the 'Submissions Needing Revision' folder to locate your manuscript file.

- A rebuttal letter that responds to each point raised by the academic editor and reviewer(s). You should upload this letter as a separate file labeled 'Response to Reviewers'.
- A marked-up copy of your manuscript that highlights changes made to the original version. You should upload this as a separate file labeled 'Revised Manuscript with Track Changes'.
- An unmarked version of your revised paper without tracked changes. You should upload this as a separate file labeled 'Manuscript'.

If you would like to make changes to your financial disclosure, please include your updated statement in your cover letter. Guidelines for resubmitting your figure files are available below the reviewer comments at the end of this letter.

If applicable, we recommend that you deposit your laboratory protocols in protocols.io to enhance the reproducibility of your results. Protocols.io assigns your protocol its own identifier (DOI) so that it can be cited independently in the future. For instructions see: https://journals.plos.org/plosone/s/submission-guidelines#loc-laboratory-protocols. Additionally, PLOS ONE offers an option for publishing peer-reviewed Lab Protocol articles, which describe protocols hosted on protocols.io. Read more information on sharing protocols at https://plos.org/protocols?utm_medium=editorial-email&utm_source=authorletters&utm_campaign=protocols.

We look forward to receiving your revised manuscript.

Kind regards,

Stefan Grosek, Ph.D., M.D.,

Academic Editor

PLOS ONE

Journal Requirements:

When submitting your revision, we need you to address these additional requirements.

1. Please ensure that your manuscript meets PLOS ONE's style requirements, including those for file naming. The PLOS ONE style templates can be found at

https://journals.plos.org/plosone/s/file?id=wjVg/PLOSOne_formatting_sample_main_body.pdf and

https://journals.plos.org/plosone/s/file?id=ba62/PLOSOne_formatting_sample_title_authors_affiliations.pdf

2. We note that the grant information you provided in the ‘Funding Information’ and ‘Financial Disclosure’ sections do not match.

When you resubmit, please ensure that you provide the correct grant numbers for the awards you received for your study in the ‘Funding Information’ section.

3. Thank you for stating the following in your manuscript:

“The authors thank the Jim Pattison Children’s Hospital Foundation. Additional support has been through NSERC, CIHR and SHRF.”

We note that you have provided funding information that is not currently declared in your Funding Statement. However, funding information should not appear in the Acknowledgments section or other areas of your manuscript. We will only publish funding information present in the Funding Statement section of the online submission form.

Please remove any funding-related text from the manuscript and let us know how you would like to update your Funding Statement. Currently, your Funding Statement reads as follows:

“The author(s) received no specific funding for this work.”

Please include your amended statements within your cover letter; we will change the online submission form on your behalf.

4. We note that your Data Availability Statement is currently as follows:

“All relevant data are within the manuscript and its Supporting Information files.”

Please confirm at this time whether or not your submission contains all raw data required to replicate the results of your study. Authors must share the “minimal data set” for their submission. PLOS defines the minimal data set to consist of the data required to replicate all study findings reported in the article, as well as related metadata and methods (https://journals.plos.org/plosone/s/data-availability#loc-minimal-data-set-definition).

For example, authors should submit the following data:

- The values behind the means, standard deviations and other measures reported;

- The values used to build graphs;

- The points extracted from images for analysis.

Authors do not need to submit their entire data set if only a portion of the data was used in the reported study.

If your submission does not contain these data, please either upload them as Supporting Information files or deposit them to a stable, public repository and provide us with the relevant URLs, DOIs, or accession numbers. For a list of recommended repositories, please see https://journals.plos.org/plosone/s/recommended-repositories.

If there are ethical or legal restrictions on sharing a de-identified data set, please explain them in detail (e.g., data contain potentially sensitive information, data are owned by a third-party organization, etc.) and who has imposed them (e.g., an ethics committee). Please also provide contact information for a data access committee, ethics committee, or other institutional body to which data requests may be sent. If data are owned by a third party, please indicate how others may request data access.

5. When completing the data availability statement of the submission form, you indicated that you will make your data available on acceptance. We strongly recommend all authors decide on a data sharing plan before acceptance, as the process can be lengthy and hold up publication timelines. Please note that, though access restrictions are acceptable now, your entire data will need to be made freely accessible if your manuscript is accepted for publication. This policy applies to all data except where public deposition would breach compliance with the protocol approved by your research ethics board. If you are unable to adhere to our open data policy, please kindly revise your statement to explain your reasoning and we will seek the editor's input on an exemption. Please be assured that, once you have provided your new statement, the assessment of your exemption will not hold up the peer review process.

6. Your ethics statement should only appear in the Methods section of your manuscript. If your ethics statement is written in any section besides the Methods, please delete it from any other section.

7. Please include a copy of Table 3 which you refer to in your text on pages 14 and 19.

8. If the reviewer comments include a recommendation to cite specific previously published works, please review and evaluate these publications to determine whether they are relevant and should be cited. There is no requirement to cite these works unless the editor has indicated otherwise.

**Additional Editor Comments:**

Dear Authors

This is interesting sstudy reviewed by two reviewers who addressed many issues to be answered by the Authors, small number of control patients, lack ofsamples in control group, and so on. From my point of view I agree very much with the reviewer, hovwever I miss date and number of approved study from the Ethics committe. The authors should explain why from collected samples of urine elapsed many years befor they collected urine samples for control group of patiients.

Refrences are missing on urine metabolomics in infants with HIE already published and which are of relevance.

Demographic data of maters and children are scarce, no informations whether induced hypothermia means wholew body hypothermia or only hypothermia of the head, ...

Kind regards

[Note: HTML markup is below. Please do not edit.]

Reviewers' comments:

Reviewer's Responses to Questions

**Comments to the Author**

1. Is the manuscript technically sound, and do the data support the conclusions?

Reviewer #1: Partly

Reviewer #2: Partly

**********

2. Has the statistical analysis been performed appropriately and rigorously?

Reviewer #1: Yes

Reviewer #2: No

**********

3. Have the authors made all data underlying the findings in their manuscript fully available?

The PLOS Data policy

Reviewer #1: Yes

Reviewer #2: Yes

**********

4. Is the manuscript presented in an intelligible fashion and written in standard English?

Reviewer #1: No

Reviewer #2: Yes

**********

Reviewer #1: The study of et Darryl al. is very interesting and potentially usefull for neonatal practice. Nevertheless there are important issues that have to be resolved before publication:

First of all the sample of the control patients is really to small: I don’t think OPLS-DA analysis are accurate for less that 5 determinations. Furthermore, In the study the eleven infants studied are classified as moderate or severe HIE. This score should be used to correctly assess the presence of HIE even if symptoms of HIE are absent or mild. The severe cases are easily identifiable. Should these patient be removed from the analysis as they may influence a score that is needed to identify potential patients that need to be treated despite having mild symptoms?

Here are other concerns that have to be addressed:

What is the goal of the study? This has to be added at the end of the introduction

The methodology that was followed is not clear, this chapter has to be written so that the reader can understand which metabolites have been tested

MS metabolomic analysis identifies significant differences in the urine of HIE infants compared to healthy infants. This paragraph is very confusing and has to be rewritten. For example are only the 15 significative metabolites being tested or all the 39?

“To refine the model, we analyzed the urine samples from the same HIE infants 2 days later, after therapeutic hypothermia treatment (n=11), which were not included in the model.” Sentence is not clear. Rephrase!

“We also analyzed the concentrations from HIE infants on day 2 of recovery, which were not used in the initial modeling (Figure 1). These metabolites suggested an intermediate metabolic phenotype between HIE and healthy infants.” Hat does “HIE infants on day 2 of recovery” mean? The sentence is really difficult to understand

What is the value of testing again the patients that have undergone hypothermia? What do the authors want to prove? This is not clear in the text

The last sentence of the conclusion has to be removed “The metabolomic-test data may also indicate the need for the involvement of a neonatologist”

Reviewer #2: The paper presents an interesting study based on urinary metabolomic analysis in a cohort of neonates with hypoxic-ischemic encephalopathy (HIE) and a small cohort of healthy controls, with the aim of identifying potential metabolites or metabolic pathways that may be pathognomonic of HIE.

Below are my observations for the Authors:

Lines 75–77: The authors correctly state that none of the methods cited, when considered individually, have predictive power with respect to HIE outcome. However, I would specify that the combination of aEEG, clinical evaluation, and metabolic acidosis may be more predictive than any of these parameters taken in isolation.

Lines 78–79: Rather than “more precise methods,” as the authors write, what is needed are methodologies capable of providing an early diagnosis, to be used alongside those currently available (clinical assessment, metabolic and lactic acidosis, and aEEG).

Line 248: The large variability in weight influences the variability of the metabolic snapshot. I would suggest describing the range of weight variability within the study limitations section.

Lines 251–252: The authors do not provide a 1:1 match between HIE patients (n=11) and healthy controls (n=4). Furthermore, the average weight of the healthy controls is not specified. I am concerned that this limits the robustness of the findings, and I recommend addressing this issue in the limitations.

Other remarks

The sample size is not large, and this should be acknowledged among the study’s limitations.

Table 2: Why were the second and third urine samples not collected in healthy neonates? It would have been interesting and useful to compare the metabolic trajectory of healthy neonates with that of those affected by HIE. Previous studies (including this, which I recommend to cite: Valerio E, Mardegan V, Stocchero M, Cavicchiolo ME, Pirillo P, Poloniato G, D'Onofrio G, Bonadies L, Giordano G, Baraldi E. Urinary metabotypes of newborns with perinatal asphyxia undergoing therapeutic hypothermia. PLoS One. 2022 Aug 16;17(8):e0273175) have shown that the metabolome of HIE neonates tends to converge with that of healthy neonates toward the end (and after the conclusion) of therapeutic hypothermia. However, without these corresponding timepoints in healthy neonates, this convergence is difficult to demonstrate in the present study.

In the Methods section, it is unclear how the authors classified a subgroup of neonates with mild HIE. Is this subclassification truly useful? The key message to convey to readers should be that metabolomics can distinguish an HIE neonate (who therefore requires therapeutic hypothermia) from a non-HIE neonate (who does not). I am not convinced that the study provides sufficient evidence to suggest that metabolomics can also be used to grade HIE severity, particularly since this would not alter the clinical management (i.e., initiation of hypothermia).

The authors do not clarify how they classified the annotation levels of the metabolites. What level of confidence do they have that the 15 identified metabolites are indeed correctly annotated? Are they all level 1 annotations? Please specify.

The data on lysine (see Fig. 2) are noteworthy. Intact lysine has been widely demonstrated to exert neuroprotective effects, both in animal models and in humans (as a reference: Valerio E, Stocchero M, Pirillo P, D'Errico I, Bonadies L, Galderisi A, Giordano G, Baraldi E. Neurosteroid pathway derangement in asphyctic infants treated with hypothermia: an untargeted metabolomic approach. EBioMedicine. 2023 Jun;92:104636. ). I would emphasize this aspect more strongly in the Discussion. Moreover, do the authors have evidence of increased lysine catabolites (e.g., pipecolic acid) in HIE neonates compared to non-HIE neonates?

The authors do not specify whether confounding factors that may interfere with metabolomic analysis (e.g., enteral/parenteral nutrition, plasma transfusions, antibiotic therapy) were present during sample collection, nor how these factors were adjusted for in the analysis. One of the major challenges of metabolomic analysis in neonates is precisely to disentangle the effects of the pathological process under investigation from those of such confounders. A comment from the authors on this point would be valuable.

**********

what does this mean?). If published, this will include your full peer review and any attached files.

If you choose “no”, your identity will remain anonymous but your review may still be made public.

**Do you want your identity to be public for this peer review?**  For information about this choice, including consent withdrawal, please see our Privacy Policy

Reviewer #1: **Yes:**  Fiammetta Piersigilli

Reviewer #2: No

**********

[NOTE: If reviewer comments were submitted as an attachment file, they will be attached to this email and accessible via the submission site. Please log into your account, locate the manuscript record, and check for the action link "View Attachments". If this link does not appear, there are no attachment files.]

While revising your submission, please upload your figure files to the Preflight Analysis and Conversion Engine (PACE) digital diagnostic tool, https://pacev2.apexcovantage.com/. PACE helps ensure that figures meet PLOS requirements. To use PACE, you must first register as a user. Registration is free. Then, login and navigate to the UPLOAD tab, where you will find detailed instructions on how to use the tool. If you encounter any issues or have any questions when using PACE, please email PLOS at figures@plos.org. Please note that Supporting Information files do not need this step.

# Author comment: Author response to Decision Letter 1

**DOI:** 10.1371/journal.pone.0361619.r002

26 Nov 2025

please see the attached Response document

Attachment Submitted filename: Response.docx

# Peer review: Decision Letter 1

**DOI:** 10.1371/journal.pone.0361619.r003

**Authors:** Stefan Grosek

22 Sep 2026

Dear Dr. Adamko,

Thank you for submitting your manuscript to PLOS One. After careful consideration, we feel that it has merit but does not fully meet PLOS One’s publication criteria as it currently stands. Therefore, we invite you to submit a revised version of the manuscript that addresses the points raised during the review process.

==============================

**ACADEMIC EDITOR:**

Please submit your revised manuscript by Nov 06 2026 11:59PM. If you will need more time than this to complete your revisions, please reply to this message or contact the journal office at plosone@plos.org. When you're ready to submit your revision, log on to https://www.editorialmanager.com/pone/ and select the 'Submissions Needing Revision' folder to locate your manuscript file.

- A letter that responds to each point raised by the academic editor and reviewer(s). You should upload this letter as a separate file labeled 'Response to Reviewers'.
- A marked-up copy of your manuscript that highlights changes made to the original version. You should upload this as a separate file labeled 'Revised Manuscript with Track Changes'.
- An unmarked version of your revised paper without tracked changes. You should upload this as a separate file labeled 'Manuscript'.

If you would like to make changes to your financial disclosure, please include your updated statement in your cover letter. Guidelines for resubmitting your figure files are available below the reviewer comments at the end of this letter.

If applicable, we recommend that you deposit your laboratory protocols in protocols.io to enhance the reproducibility of your results. Protocols.io assigns your protocol its own identifier (DOI) so that it can be cited independently in the future. For instructions see: https://journals.plos.org/plosone/s/submission-guidelines#loc-laboratory-protocols. Additionally, PLOS One offers an option for publishing peer-reviewed Lab Protocol articles, which describe protocols hosted on protocols.io. Read more information on sharing protocols at https://plos.org/protocols?utm_medium=editorial-email&utm_source=authorletters&utm_campaign=protocols.

As the corresponding author, your ORCID iD is verified in the submission system and will appear in the published article. PLOS supports the use of ORCID, and we encourage all coauthors to register for an ORCID iD and use it as well. Please encourage your coauthors to verify their ORCID iD within the submission system before final acceptance, as unverified ORCID iDs will not appear in the published article. *Only*  the individual author can complete the verification step; PLOS staff *cannot*  verify ORCID iDs on behalf of authors.

We look forward to receiving your revised manuscript.

Kind regards,

Stefan Grosek, Ph.D., M.D.,

Academic Editor

PLOS One

Journal Requirements:

If the reviewer comments include a recommendation to cite specific previously published works, please review and evaluate these publications to determine whether they are relevant and should be cited. There is no requirement to cite these works unless the editor has indicated otherwise.

Additional Editor Comments:

Dear Authors

Despite much improvement in revised version of your article the third reviewer found some important issues to be addressed before final decision: The principal issue is that the reported “100% accuracy” does not appear to represent an independent validation: the day-3 samples from the same HIE infants were used during metabolite model refinement were also used as longitudinal samples subsequently described as a blinded test set. Given the very small dataset, the use of 15 metabolites and seven OPLS-DA components, there is a substantial risk of overfitting. The analysis would be more convincing with nested cross-validation/permutation testing and diagnostic measures such as sensitivity, specificity, and AUC with confidence intervals, followed ultimately by validation in an independent cohort.

A second concern is potential confounding: HIE samples were collected in 2018-2020 whereas healthy controls were recruited in 2023-2024, creating possible storage-duration, batch, and temporal differences that are not adequately addressed. Additionally, statements that the metabolomic approach is superior to lactate, more accurate than EEG/MRI, or can identify infants at risk of long-term neurologic injury are stronger than can be supported by this small pilot study without an independent validation cohort or long-term neurologic outcomes. I would therefore encourage further revision, particularly to tone down the conclusions and more explicitly separate the proof-of-concept results from validated diagnostic performance.

[Note: HTML markup is below. Please do not edit.]

Reviewers' comments:

Reviewer's Responses to Questions

**Comments to the Author**

Reviewer #2: All comments have been addressed

Reviewer #3: (No Response)

**********

2. Is the manuscript technically sound, and do the data support the conclusions?

Reviewer #2: Yes

Reviewer #3: Partly

**********

3. Has the statistical analysis been performed appropriately and rigorously?

Reviewer #2: Yes

Reviewer #3: Yes

**********

4. Have the authors made all data underlying the findings in their manuscript fully available?

The PLOS Data policy

Reviewer #2: Yes

Reviewer #3: Yes

**********

5. Is the manuscript presented in an intelligible fashion and written in standard English?

Reviewer #2: Yes

Reviewer #3: Yes

**********

Reviewer #2: (No Response)

Reviewer #3: The revised manuscript is clearer and the authors have addressed several reviewer concerns, including clarification of the study objective, expansion of the limitations and addition of relevant prior literature. However, important methodological concerns remain. The principal issue is that the reported “100% accuracy” does not appear to represent an independent validation: the day-3 samples from the same HIE infants were used during metabolite model refinement were also used as longitudinal samples subsequently described as a blinded test set. Given the very small dataset, the use of 15 metabolites and seven OPLS-DA components, there is a substantial risk of overfitting. The analysis would be more convincing with nested cross-validation/permutation testing and diagnostic measures such as sensitivity, specificity, and AUC with confidence intervals, followed ultimately by validation in an independent cohort.

A second concern is potential confounding: HIE samples were collected in 2018-2020 whereas healthy controls were recruited in 2023-2024, creating possible storage-duration, batch, and temporal differences that are not adequately addressed. Additionally, statements that the metabolomic approach is superior to lactate, more accurate than EEG/MRI, or can identify infants at risk of long-term neurologic injury are stronger than can be supported by this small pilot study without an independent validation cohort or long-term neurologic outcomes. I would therefore encourage further revision, particularly to tone down the conclusions and more explicitly separate the proof-of-concept results from validated diagnostic performance.

**********

what does this mean?). If published, this will include your full peer review and any attached files.

If you choose “no”, your identity will remain anonymous but your review may still be made public.

**Do you want your identity to be public for this peer review?**  For information about this choice, including consent withdrawal, please see our Privacy Policy

Reviewer #2: No

Reviewer #3: No

**********

[NOTE: If reviewer comments were submitted as an attachment file, they will be attached to this email and accessible via the submission site. Please log into your account, locate the manuscript record, and check for the action link "View Attachments". If this link does not appear, there are no attachment files.]

To ensure your figures meet our technical requirements, please review our figure guidelines: https://journals.plos.org/plosone/s/figures

You may also use PLOS’s free figure tool, NAAS, to help you prepare publication quality figures: https://journals.plos.org/plosone/s/figures#loc-tools-for-figure-preparation.

NAAS will assess whether your figures meet our technical requirements by comparing each figure against our figure specifications.

# Author comment: Author response to Decision Letter 2

**DOI:** 10.1371/journal.pone.0361619.r004

30 Sep 2026

Dear Dr. Grosek,

Thank you for the opportunity to submit a revised version of the manuscript using track changes and the point by point responses to the Reviewers below.

Editor Comments:

Dear Authors

Despite much improvement in revised version of your article the third reviewer found some important issues to be addressed before final decision: The principal issue is that the reported “100% accuracy” does not appear to represent an independent validation: the day-3 samples from the same HIE infants were used during metabolite model refinement were also used as longitudinal samples subsequently described as a blinded test set. Given the very small dataset, the use of 15 metabolites and seven OPLS-DA components, there is a substantial risk of overfitting. The analysis would be more convincing with nested cross-validation/permutation testing and diagnostic measures such as sensitivity, specificity, and AUC with confidence intervals, followed ultimately by validation in an independent cohort.

A second concern is potential confounding: HIE samples were collected in 2018-2020 whereas healthy controls were recruited in 2023-2024, creating possible storage-duration, batch, and temporal differences that are not adequately addressed. Additionally, statements that the metabolomic approach is superior to lactate, more accurate than EEG/MRI, or can identify infants at risk of long-term neurologic injury are stronger than can be supported by this small pilot study without an independent validation cohort or long-term neurologic outcomes. I would therefore encourage further revision, particularly to tone down the conclusions and more explicitly separate the proof-of-concept results from validated diagnostic performance.

Response: We understand the Editor’s points and have addressed them below to the specific Reviewer.

Comments to the Author

1. If the authors have adequately addressed your comments raised in a previous round of review and you feel that this manuscript is now acceptable for publication, you may indicate that here to bypass the “Comments to the Author” section, enter your conflict of interest statement in the “Confidential to Editor” section, and submit your "Accept" recommendation.

Reviewer #2: All comments have been addressed

Response: Thank you

Reviewer #3: (No Response)

________________________________________

2. Is the manuscript technically sound, and do the data support the conclusions?

The manuscript must describe a technically sound piece of scientific research with data that supports the conclusions. Experiments must have been conducted rigorously, with appropriate controls, replication, and sample sizes. The conclusions must be drawn appropriately based on the data presented.

Reviewer #2: Yes

Reviewer #3: Partly

Response: Thank you

________________________________________

3. Has the statistical analysis been performed appropriately and rigorously?

Reviewer #2: Yes

Reviewer #3: Yes

Response: Thank you

________________________________________

4. Have the authors made all data underlying the findings in their manuscript fully available?

The PLOS Data policy requires authors to make all data underlying the findings described in their manuscript fully available without restriction, with rare exception (please refer to the Data Availability Statement in the manuscript PDF file). The data should be provided as part of the manuscript or its supporting information, or deposited to a public repository. For example, in addition to summary statistics, the data points behind means, medians and variance measures should be available. If there are restrictions on publicly sharing data—e.g. participant privacy or use of data from a third party—those must be specified.

Reviewer #2: Yes

Reviewer #3: Yes

Response: Thank you

________________________________________

5. Is the manuscript presented in an intelligible fashion and written in standard English?

PLOS ONE does not copyedit accepted manuscripts, so the language in submitted articles must be clear, correct, and unambiguous. Any typographical or grammatical errors should be corrected at revision, so please note any specific errors here.

Reviewer #2: Yes

Reviewer #3: Yes

Response: Thank you

________________________________________

6. Review Comments to the Author

Please use the space provided to explain your answers to the questions above. You may also include additional comments for the author, including concerns about dual publication, research ethics, or publication ethics. (Please upload your review as an attachment if it exceeds 20,000 characters)

Reviewer #2: (No Response)

Response: Thank you

Reviewer #3:

1. The revised manuscript is clearer and the authors have addressed several reviewer concerns, including clarification of the study objective, expansion of the limitations and addition of relevant prior literature.

Response: Thank you

2. However, important methodological concerns remain. The principal issue is that the reported “100% accuracy” does not appear to represent an independent validation: the day-3 samples from the same HIE infants were used during metabolite model refinement were also used as longitudinal samples subsequently described as a blinded test set. Given the very small dataset, the use of 15 metabolites and seven OPLS-DA components, there is a substantial risk of overfitting. The analysis would be more convincing with nested cross-validation/permutation testing and diagnostic measures such as sensitivity, specificity, and AUC with confidence intervals, followed ultimately by validation in an independent cohort.

Response: We acknowledge this is a small data set. The study struggled to recruit because of the COVID outbreak in 2020. We have added that text to the Limitations section. We also agree that the “accuracy” statements need to be changed. The Day 3 samples we not truly blinded but instead represent a method to remove metabolites of low importance but also over-fitting (line 275). We agree that the goal is to avoid over-fitting. We have also added the Reviewer’s statistical suggestions for a future study in the Limitations section (line 393). We hope the Reviewer can now see the value of these data to publish. We are in the process of writing grants based on the pilot data and a publication is essential. Thank you.

3. A second concern is potential confounding: HIE samples were collected in 2018-2020 whereas healthy controls were recruited in 2023-2024, creating possible storage-duration, batch, and temporal differences that are not adequately addressed.

Response: The time delay occurred as a result of the COVID outbreak. We have stated this openly in the manuscript, and we have stated this as a limitation of the study in the Discussion line 388. We hope this will satisfy the Reviewer.

4. Additionally, statements that the metabolomic approach is superior to lactate, more accurate than EEG/MRI, or can identify infants at risk of long-term neurologic injury are stronger than can be supported by this small pilot study without an independent validation cohort or long-term neurologic outcomes. I would therefore encourage further revision, particularly to tone down the conclusions and more explicitly separate the proof-of-concept results from validated diagnostic performance.

Response: We agree with the Reviewer and we have made extensive changes in the text throughout the marked manuscript (Abstract, Introduction, and Discussion) to reflect the pilot nature of these data. We are not saying that this “is” superior to Lactate alone (or MRI/EEG), but this “could be”. We also have clearly stated the limitations in the Discussion as suggested. This paper should be interesting to clinicians. I hope we have satisfied the Reviewer with this text. Thank you.

Attachment Submitted filename: Response2 Plos.docx

# Peer review: Decision Letter 2

**DOI:** 10.1371/journal.pone.0361619.r005

**Authors:** Stefan Grosek

13 Oct 2026

Targeted Urinary Metabolomic Profiling in Newborns Undergoing Therapeutic Hypothermia for Hypoxic-Ischemic Encephalopathy: A Pilot Study

PONE-D-25-25931R2

Dear Dr. Adamko,

We’re pleased to inform you that your manuscript has been judged scientifically suitable for publication and will be formally accepted for publication once it meets all outstanding technical requirements.

Within one week, you’ll receive an e-mail detailing the required amendments. When these have been addressed, you’ll receive a formal acceptance letter and your manuscript will be scheduled for publication.

An invoice will be generated when your article is formally accepted. Please note, if your institution has a publishing partnership with PLOS and your article meets the relevant criteria, all or part of your publication costs will be covered. Please make sure your user information is up-to-date by logging into Editorial Manager at Editorial Manager® and clicking the ‘Update My Information' link at the top of the page. For questions related to billing, please contact billing support.

If your institution or institutions have a press office, please notify them about your upcoming paper to help maximize its impact. If they’ll be preparing press materials, please inform our press team as soon as possible -- no later than 48 hours after receiving the formal acceptance. Your manuscript will remain under strict press embargo until 2 pm Eastern Time on the date of publication. For more information, please contact onepress@plos.org.

Kind regards,

Stefan Grosek, Ph.D., M.D.,

Academic Editor

PLOS One

Additional Editor Comments (optional):

Reviewers' comments:

Reviewer's Responses to Questions

**Comments to the Author**

Reviewer #3: (No Response)

**********

2. Is the manuscript technically sound, and do the data support the conclusions?

Reviewer #3: Yes

**********

3. Has the statistical analysis been performed appropriately and rigorously?

Reviewer #3: Yes

**********

4. Have the authors made all data underlying the findings in their manuscript fully available?

The PLOS Data policy

Reviewer #3: Yes

**********

5. Is the manuscript presented in an intelligible fashion and written in standard English?

Reviewer #3: Yes

**********

Reviewer #3: all comments addressed..there are no additional comments and I appreciate the authors' inclusion of limitations for a more accurate representation of their findings

**********

what does this mean?). If published, this will include your full peer review and any attached files.

If you choose “no”, your identity will remain anonymous but your review may still be made public.

**Do you want your identity to be public for this peer review?**  For information about this choice, including consent withdrawal, please see our Privacy Policy

Reviewer #3: No

**********

# Editor report: Acceptance letter

**DOI:** 10.1371/journal.pone.0361619.r006

**Authors:** Stefan Grosek

PONE-D-25-25931R2

PLOS One

Dear Dr. Adamko,

I'm pleased to inform you that your manuscript has been deemed suitable for publication in PLOS One. Congratulations! Your manuscript is now being handed over to our production team.

At this stage, our production department will prepare your paper for publication. This includes ensuring the following:

* All references, tables, and figures are properly cited

* All relevant supporting information is included in the manuscript submission,

* There are no issues that prevent the paper from being properly typeset

You will receive further instructions from the production team, including instructions on how to review your proof when it is ready. Please keep in mind that we are working through a large volume of accepted articles, so please give us a few days to review your paper and let you know the next and final steps.

Lastly, if your institution or institutions have a press office, please let them know about your upcoming paper now to help maximize its impact. If they'll be preparing press materials, please inform our press team within the next 48 hours. Your manuscript will remain under strict press embargo until 2 pm Eastern Time on the date of publication. For more information, please contact onepress@plos.org.

You will receive an invoice from PLOS for your publication fee after your manuscript has reached the completed accept phase. If you receive an email requesting payment before acceptance or for any other service, this may be a phishing scheme. Learn how to identify phishing emails and protect your accounts at https://explore.plos.org/phishing.

If we can help with anything else, please email us at customercare@plos.org.

Thank you for submitting your work to PLOS One and supporting open access.

Kind regards,

PLOS One Editorial Office Staff

on behalf of

Professor Stefan Grosek

Academic Editor

PLOS One
